# Neutrophil-derived reactive agents induce a transient SpeB negative phenotype in *Streptococcus pyogenes*

**DOI:** 10.1186/s12929-023-00947-x

**Published:** 2023-07-10

**Authors:** Patience Shumba, Thomas Sura, Kirsten Moll, Bhavya Chakrakodi, Lea A. Tölken, Jörn Hoßmann, Katharina J. Hoff, Ole Hyldegaard, Michael Nekludov, Mattias Svensson, Per Arnell, Steinar Skrede, INFECT Study Group, Morten Hedetoft, Trond Bruun, Oddvar Oppegaard, Torbjørn Nedrebø, Eivind Rath, Martin Bruun Madsen, Anna Norrby-Teglund, Nikolai Siemens

**Affiliations:** 1 Department of Molecular Genetics and Infection Biology, University of Greifswald, Greifswald, Germany; 2 Department of Microbial Proteomics, Institute of Microbiology, University of Greifswald, Greifswald, Germany; 3 Center for Infectious Medicine, Karolinska Institutet, Karolinska University Hospital, Huddinge, Stockholm, Sweden; 4 Helmholtz Center for Infection Research, Brunswick, Germany; 5 Institute of Mathematics and Computer Science, University of Greifswald, Greifswald, Germany; 6 Department of Anaesthesia, Head and Orthopedic Center, University Hospital Copenhagen, Rigshospitalet, Copenhagen, Denmark; 7 Institute of Clinical Medicine, University of Copenhagen, Copenhagen, Denmark; 8 Department of Anaesthesia, Surgical Services and Intensive Care, Karolinska Institute, Karolinska University Hospital, Stockholm, Sweden; 9 Department of Anaesthesiology and Intensive Care Medicine, Sahlgrenska University Hospital, Gothenburg, Sweden; 10 Department of Medicine, Haukeland University Hospital, Bergen, Norway; 11 Department of Clinical Science, University of Bergen, Bergen, Norway

**Keywords:** *Streptococcus pyogenes*, Necrotizing soft tissue infections, SpeB, Neutrophils

## Abstract

**Background:**

*Streptococcus pyogenes* (group A streptococci; GAS) is the main causative pathogen of monomicrobial necrotizing soft tissue infections (NSTIs). To resist immuno-clearance, GAS adapt their genetic information and/or phenotype to the surrounding environment. Hyper-virulent streptococcal pyrogenic exotoxin B (SpeB) negative variants caused by *cov*RS mutations are enriched during infection. A key driving force for this process is the bacterial Sda1 DNase.

**Methods:**

Bacterial infiltration, immune cell influx, tissue necrosis and inflammation in patient´s biopsies were determined using immunohistochemistry. SpeB secretion and activity by GAS post infections or challenges with reactive agents were determined via Western blot or casein agar and proteolytic activity assays, respectively. Proteome of GAS single colonies and neutrophil secretome were profiled, using mass spectrometry.

**Results:**

Here, we identify another strategy resulting in SpeB-negative variants, namely reversible abrogation of SpeB secretion triggered by neutrophil effector molecules. Analysis of NSTI patient tissue biopsies revealed that tissue inflammation, neutrophil influx, and degranulation positively correlate with increasing frequency of SpeB-negative GAS clones. Using single colony proteomics, we show that GAS isolated directly from tissue express but do not secrete SpeB. Once the tissue pressure is lifted, GAS regain SpeB secreting function. Neutrophils were identified as the main immune cells responsible for the observed phenotype. Subsequent analyses identified hydrogen peroxide and hypochlorous acid as reactive agents driving this phenotypic GAS adaptation to the tissue environment. SpeB-negative GAS show improved survival within neutrophils and induce increased degranulation.

**Conclusions:**

Our findings provide new information about GAS fitness and heterogeneity in the soft tissue milieu and provide new potential targets for therapeutic intervention in NSTIs.

**Supplementary Information:**

The online version contains supplementary material available at 10.1186/s12929-023-00947-x.

## Introduction

Necrotizing soft tissue infections (NSTIs) are rapidly progressing infections of any layer of the skin or soft tissue. The infections are associated with significant morbidity and mortality [22, 39]. Extensive surgical interventions and even amputations are often required despite intensive care and prompt antibiotic therapy [1]. Based on the microbiological etiology, NSTIs are categorized into two main types: type 1 of polymicrobial and type 2 of monomicrobial nature [37]. *Streptococcus pyogenes* (group A streptococci [GAS]) is the major causative pathogen of NSTIs [3, 22]. GAS NSTIs are more frequent among younger individuals without comorbidities, preferably located to the extremities, and often complicated by streptococcal toxic shock syndrome (STSS) [4, 22, 41]. A recent Scandinavian prospective multicenter study called INFECT enrolled 409 surgically confirmed NSTI cases, among which 31% constituted GAS cases [4, 22].

Several studies have shown that GAS NSTIs are characterized by high bacterial load and a hyper-inflammatory response characterized by a massive infiltration of immune cells, including neutrophils and macrophages, and elevated levels of pro-inflammatory molecules [14, 15, 26, 27, 33, 42]. The ability of GAS to cause hyper-inflammation and tissue damage can be attributed to an arsenal of virulence factors. In light of neutrophil activation, several GAS virulence factors such as M-protein and phosphoglycerate kinase (PGK) have been identified as potent inducers of neutrophil activation and degranulation resulting in release of proteolytic enzymes and other effector molecules into the surrounding milieu and subsequent tissue damage [14, 15, 32, 33, 35, 38, 43]. Another important virulence factor is the streptococcal pyrogenic exotoxin B (SpeB), which is a cysteine protease targeting numerous important host substrates such as immunoglobulins, complement, antimicrobial peptides, and interleukin (IL)-1β [25, 37]. Notably, it also has endogenous substrates including several important virulence factors [37]. In the case of PGK, neutrophil activation is only evident in SpeB-negative variants [43]. Such variants have been ascribed hyper-virulent properties and reported to be enriched during infection through mutations in genes encoding CovR/S two-component system and stand-alone regulator RopB resulting in an irreversible loss of SpeB expression [13, 40].

Bacterial killing by neutrophils involves both non-oxidative and oxidative mechanisms. The latter of which is ensured through reactive oxygen species (ROS). ROS are generated via the NADPH oxidase and myeloperoxidase (MPO) systems [47]. Hydrogen peroxide (H_2_O_2_) is produced by spontaneous or dismutase driven conversion of superoxide. The subsequent MPO-catalysed oxidation of chlorine by H_2_O_2_ results in formation of hypochlorous acid (HOCl) [24]. MPO is highly abundant within azurophilic granules of neutrophils. Hence, HOCl can be found intracellularly within neutrophils as well as extracellularly [28].

The role of SpeB in NSTI remains elusive. In our previous report [33], we identified that NSTI patient tissue biopsies frequently contained a mixture of SpeB-positive (SpeB^+^) and SpeB-negative (SpeB^−^) clones as assessed by proteolytic assays. In this study, we further characterized these clones and through single colony proteomics, we show that the bacteria express but do not secrete SpeB. We further show that this phenotype is induced by the neutrophils, and particularly MPO-derived HOCl and its precursor, H_2_O_2_, resulting in SpeB-negative GAS clones that survive and multiply within neutrophils, induce excessive degranulation, and thereby contribute to tissue disruptive and hyper-inflammatory processes in NSTIs.

## Methods

### Bacterial strains

GAS 5448, 5626, 8003, and 8157 are NSTI isolates from Toronto, Canada (provided by Donald E. Low, Mount Sinai Hospital, Toronto, Canada) [16, 19]. All strains were cultured in Todd-Hewitt broth (Carl Roth) supplemented with 1.5% (w/v) yeast extract (Carl Roth) at 37 °C. GAS strains from the INFECT NSTI patients were recovered directly by culture from the tissue biopsies.

### SpeB expression and protease activity analyses

The SpeB protease activity was determined as described previously [34]. Briefly, the bacteria were grown in THY (16–18 h), centrifuged, the supernatants were collected and filtered (pore size, 0.20 µm). Supernatants were incubated with 5 mM DTT for 30 min at 37 °C, after which *n*-benzoyl-proline-phenylalanine-arginine-*p*-nitroanilide hydrochloride (1 mM) and phosphate buffer (pH 6; 5 mM) were added and absorbance at 405 nm was detected. Experiments also included testing activity with addition of H_2_O_2_ (10 and 100 µM). Furthermore, SpeB secretion and activity were tested via casein digestion assay as described previously [21]. Serial dilutions of bacteria were plated on modified Columbia agar containing 3% (w/v) skim milk (both Sigma-Aldrich) following incubation under 37 °C and 5% CO_2_ atmosphere for 24 h. SpeB^+^ producers were characterized by a clearance zone around the colonies, whereas non-producers had no zone of clearance.

For Western blot analyses, bacterial supernatants were precipitated with 96% (v/v) ethanol over night at − 20 °C. Equal amounts of protein were incubated with sodium dodecyl sulfate loading buffer at 90 °C for 10 min. Proteins were separated using a 4–12% SDS-PAGE gel and transferred onto a nitrocellulose membrane. The membrane was blocked with 5% (w/v) dry casein powder in 0.05% (v/v) Tween-20 Tris-buffered saline. The following antibodies were used for detection: anti-SpeB antibody (Abcam) and secondary anti-rabbit IgG horseradish peroxidase linked Fab fragment (GE Healthcare).

### Antimicrobial testing

1 × 10^6^ CFU/ml of bacterial strains were exposed to different concentrations of clindamycin, benzylpenicillin (both Sigma-Aldrich), LL-37 (Invivogen), Lysozyme (Sigma-Aldrich), HNP-1 (Peprotech), MPO, resistin, HBP (R&D Systems), and H_2_O_2_ (Sigma-Aldrich) for 3 h and plated in serial dilutions on casein agar plates. CFUs were analyzed after 24 h of incubation at 37 °C and 5% CO_2_.

### Human cells, culture conditions, and infections

The human keratinocyte cell line N/TERT-1 (a gift from J. Rheinwald and the Cell Culture Core of the Harvard Skin Disease Research Centre, Boston, MA, USA) was cultured in EpiLife medium (Invitrogen). Primary normal human dermal fibroblasts (NHDFs) were cultured in DMEM (Invitrogen) supplemented with 10% (v/v) FCS (Invitrogen). Human primary neutrophils were isolated from healthy donors using a density gradient centrifugation on Polymorphprep (Axis Shield). Neutrophil viability was assessed via trypan blue staining. After isolation, neutrophils were suspended in RPMI1640 medium (HyClone) supplemented with 10 mM l-glutamine, 25 mmol/l HEPES (all HyClone), and 5% (v/v) FCS. PBMCs were isolated from buffy coats by Lymphoprep (Axis-Shields) gradient centrifugation. Cells were allowed to adhere in cell culture flasks (Corning) for 30 min at 37 °C in serum free RPMI1640 media. The non-adherent cells were removed by washing with PBS (HyClone). Monocyte-derived macrophages were generated by culturing primary human monocytes isolated from peripheral blood of healthy donors in 6-well plates (Corning) for 9 days at a density of 1 × 10^6^ cells/well. Monocytes were differentiated to pro-inflammatory macrophages for 7 days in cell culture media containing GM-CSF (25 ng/ml), followed by two additional days of incubation with LPS (100 ng/ml). Media was changed every 2–3 days. All cells were cultured under 37 °C and 5% CO_2_ atmosphere.

All infections were performed at a multiplicity of infection (MOI) 10 in a final volume of 1 ml of the respective media. Adherence to and internalization into N/TERT-1 cells and NHDFs were quantified using the antibiotic protection assay [23]. Briefly, 24-well plates were inoculated with 2.5 × 10^5^ N/TERT-1 or NHDFs/well without antibiotics. The cells were allowed to grow to confluence. For the assay, cells were washed and infected with GAS (MOI 10). Two hours after infection, the viable counts of bacteria (colony-forming units, CFU) released from lysed cells were determined by plating on blood agar. For the assessment of bacterial internalization, 2 h after infection, the cells were washed with PBS and incubated with media supplemented with benzylpenicillin (20 μg ml^−1^) and gentamicin (120 μg ml^−1^) for additional 2 or 4 h. Subsequently, the cells were washed, lysed, and the CFU counts were determined as described above. For the assessment of SpeB release and activity, intracellular bacteria were plated on casein agar plates.

1 × 10^6^ macrophages were infected for 2 h following 1 h of antibiotic treatment and 5 × 10^5^ neutrophils were infected for 1 h following antibiotic treatment. Assessment of intracellular bacterial numbers was performed as described above. For MPO inhibition, different concentrations of 4-aminobenzoic hydrazide (Sigma-Aldrich) were used: 25, 50 and 100 µM. After isolation, neutrophils were suspended in RPMI media with MPO inhibitor and incubated at 37 °C for 30 min prior to infections. All infections were performed in the presence of the inhibitor. Bacterial survival was evaluated 1, 2 and 4 h post infections. For proteomics, all infections were performed in Hank’s balanced salts solution (HBSS, Invitrogen). Neutrophil supernatants were collected and stored at − 20 °C until analysis.

### Immunostaining and analysis of tissue biopsies

Patient biopsies were cryosectioned (5–8 μm) using a MICROM cryostat HM 560 MV (Zeiss), fixed in 2% (v/v) formaldehyde or ice-cold acetone, and immunostained as previously described [33, 35]. The following antibodies were used for immunohistochemistry: anti-human HMGB1 (clone EPR3507; Abcam), anti-human IL-8 (clone NAP-1; Invitrogen), anti-human resistin (clone 184,305; R&D systems), and anti-human neutrophil-elastase (clone NP57; DAKO). Biotinylated secondary antibodies included goat anti-mouse IgG and goat anti-rabbit IgG (both from Vector Laboratories). The immunohistochemically stained sections were analyzed by acquired computerized image analysis (ACIA) [20]. The cell area was defined by the hematoxylin counterstaining, and the results are presented as percent positively stained area × mean intensity of positive staining.

The following antibodies were used for immunofluorescence analyses: anti-MPO (MAB3174; R&D), anti-GAS (9191; Abcam). Secondary antibodies included anti-mouse IgG AF488, anti-goat IgG AF594, and anti-rabbit IgG AF594 (all Invitrogen). IF-images were acquired with Olympus BXS1 microscope and Olympus cellSense software (Olympus) and/or Leica Stellaris 8 confocal laser scanning microscope (CLSM) and LasX software (Leica Microsystems). Four to six images were taken per biopsy. Analysis of the micrographs was performed in a two-step procedure, which included an automated recognition of GAS and MPO stainings followed by a calculation of the stained area (px^2^). Each picture was manually checked and if necessary corrected after an automated recognition.

### Whole genome sequencing and data processing

DNA extraction from GAS strains 5448, 5626, 8003, and 8157, whole genome sequencing, and analyses were performed as previously described [36]. Detailed description is provided in the Additional file 1.

### Protein extraction, LC–MS/MS analyses, and data processing of single GAS colonies and neutrophil secretome

Neutrophil secretome was determined as previously described [8]. Detailed description of protein extraction, measurements, and analyses are provided in the Additional file 1.

### Statistics

If not otherwise indicated, statistical significance of differences was determined using the 2-tailed Mann–Whitney *U* test. Multiple comparisons were done using Kruskal Wallis test with Dunn’s post-test. Correlation analyses were determined using Spearman test. Statistics were performed using GraphPad Prism version 7 (GraphPad software). A *p*-value less than 0.05 was considered significant.

## Results

### Reversible loss of SpeB proteolytic activity by GAS is associated with tissue pathology and inflammation

**Fig. 1:**
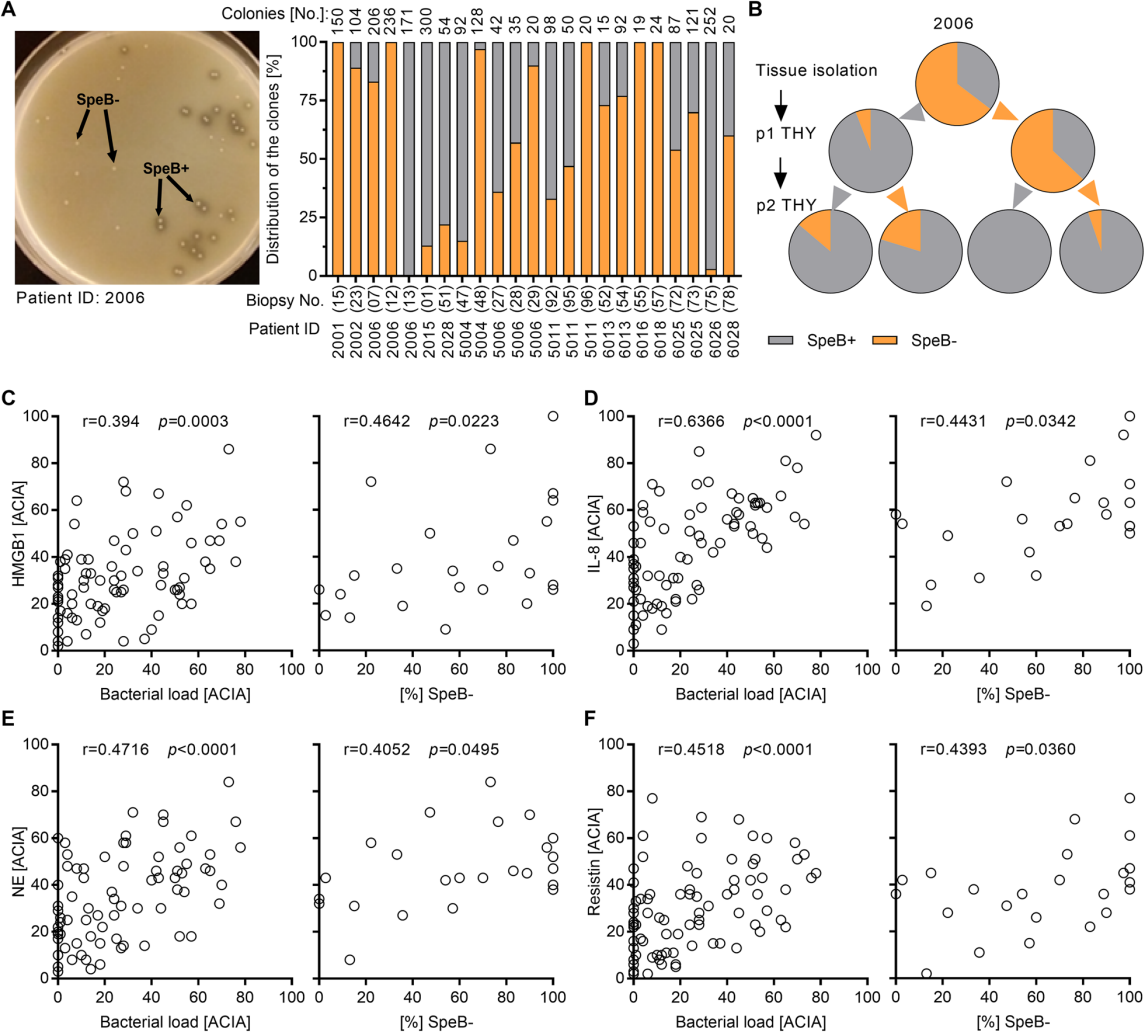
Reversible loss of SpeB is associated with tissue pathology and inflammation. **A** Distribution of SpeB^+^ and SpeB^−^ GAS clones directly isolated from NSTI patient tissue biopsies (n = 23). **B** Percentage of SpeB^+^ and SpeB^−^ GAS clones after the passage in THY media (p1, passage 1; p2, passage 2). Representative analysis of 2006 GAS patient isolate is shown. **C**–**G** Correlation analysis of bacterial load (left panel; n = 81 biopsies) or percentage of SpeB^−^ clones (right panel; n = 23 biopsies) with the presence of HMGB1 (**C**), IL-8 (**D**), infiltrating neutrophils (**E**), and resistin (**F**) in patient biopsies. Correlation was determined using Spearman test. Semiquantitative acquired computerized image analyses (ACIA) of immuno-histochemical staining were performed as described in the methods section. The cell area was defined by the hematoxylin counterstaining, and the results are presented as percent positively stained area × mean intensity of positive staining

Bacteria were isolated from tissue biopsies collected from GAS patients (Additional file 2: Table S1) through direct plating of tissue sections on casein agar plates to assess SpeB activity. These analyses confirmed previous observations that infected tissue harbors a mixture of SpeB^+^ and SpeB^−^ clones (Fig. 1A). The majority of successor generations of SpeB^−^ clones returned to a wild-type-like SpeB^+^ phenotype after cultivation in THY media (Fig. 1B and Additional file 1: Fig. S1). To confirm that the observed SpeB^−^ phenotype is not linked to genetic variations within mutational hotspots (*covS*, *covR*, and *ropB*) or *speB*, comparative whole genome sequence analyses were performed. Although strains 2001, 2006, 6016, and 6018 were exclusively SpeB^−^ post direct tissue isolation (Fig. 1A), no mutations in these strains were found. In general, the analyses of these hotspots revealed only minor variance on nucleotide and amino acid levels (Additional file 1: Fig. S2-S4). CovR amino acid sequences were 100% identical between all strains. In strain 2002, *covS* initiated with the rare start codon ATA [11] and a frame shift causing single nucleotide deletion in position 381 was detected (Additional file 1: Fig. S2). Most of the mutations were found in strain 6028, *e.g.,* mutations within *covS* leading to several amino acid substitutions and a notable deletion of 45 nucleotides (Additional file 1: Figs. S2 and S3). Furthermore, single mutations leading to amino acid substitution within RopB were found in strains 2002, 5004, 6026, and 6028 (Additional file 1: Fig. S4). *SpeB* gene itself was unaffected. Additional exemplary sequence analyses of other TCS kinases/regulators identified rather *emm*-type specific mutations resulting in conserved amino acid substitutions, which would most likely not affect SpeB expression (Additional file 2: Table S2). These results suggest that in addition to irreversible loss of SpeB expression due to mutations in *covR/S* or *ropB* regions, reversible abrogation of SpeB expression at protein or activity level occurs in the tissue setting.

The observation of heterogeneity in SpeB proteolytic activity led us to explore how this related to tissue inflammation and damage. For this purpose, biopsies collected from repeated surgeries at different sites of infection (*e.g.*, fascia, soft tissue, and muscle) of 31 GAS NSTI patients were analyzed for the presence of markers of tissue necrosis [high-mobility-group-protein B1 (HMGB1)], inflammation (IL-8), neutrophil infiltration [neutrophil elastase (NE)], and neutrophil degranulation (resistin; Additional file 1: Fig. S5). Correlation analyses based on bacterial load and the percentage of SpeB^−^ clones recovered from the respective tissue specimen with each of the markers were performed. All of the analyzed markers showed a positive correlation to bacterial load (Fig. 1C–F). Moreover, positive correlations between tissue necrosis, inflammation, and neutrophil infiltration as well as degranulation with increasing percentage of SpeB^−^ clones, irrespective of their rise through genetic or phenotypic variations, were found (Fig. 1C–F).

### Intracellular GAS recovered from neutrophils show reversible loss of SpeB proteolytic activity

**Fig. 2:**
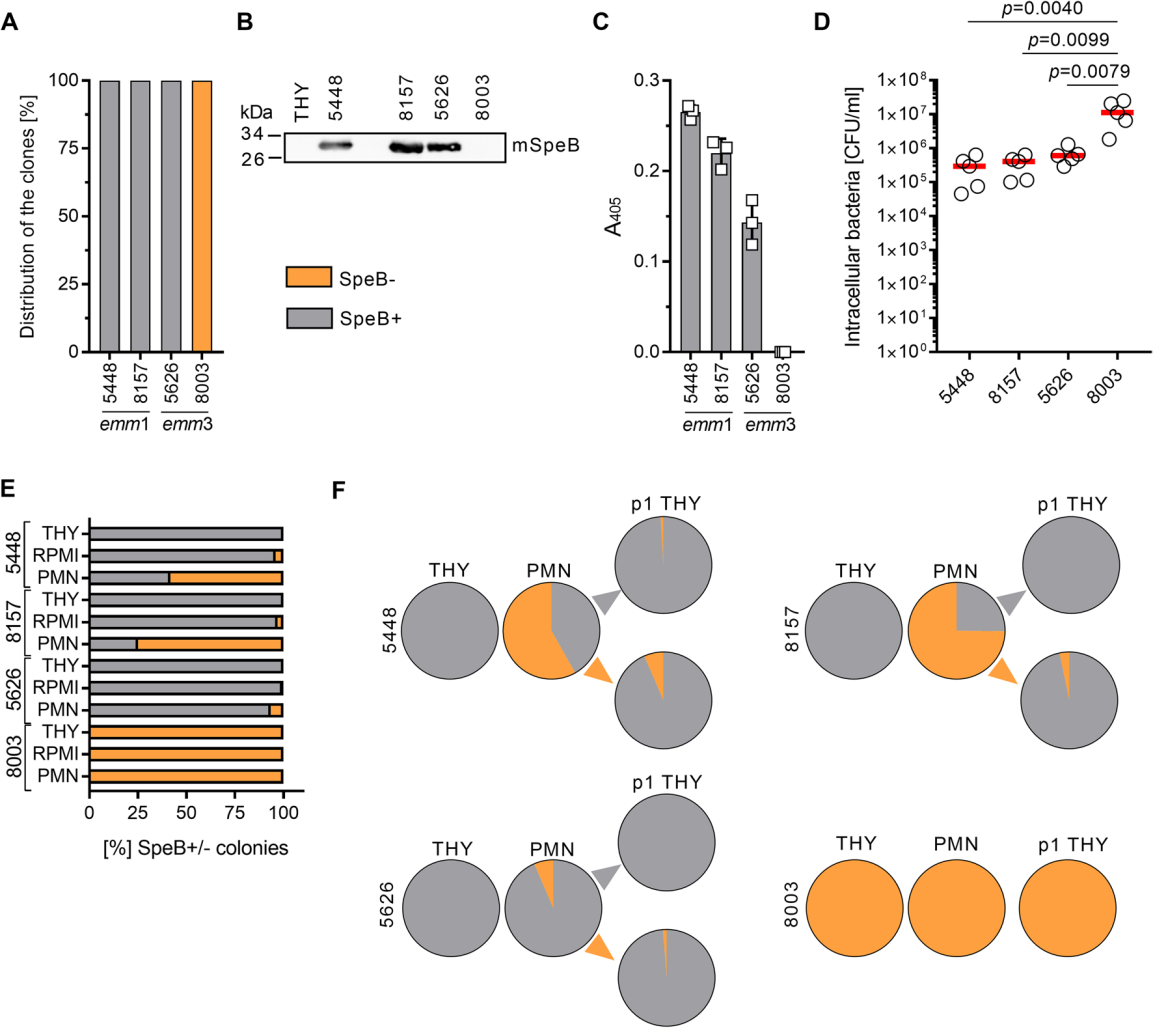
Human neutrophils induce loss of SpeB in GAS. **A** Assessment of SpeB positivity/negativity of indicated strains via casein agar assay. Mean percentage from four independent experiments is shown (n = 4). **B** Representative image of *Western*-Blot analyses (n = 3; mSpeB, mature SpeB) and (**C**) SpeB activity assay (n = 3) of bacterial supernatants. GAS were grown over-night (16 h) in THY and sterile-filtered supernatants were used for the analyses. Original blot is shown in Additional file 1: Fig. S8. Each dot in **C** represents one independent experiment. Bars denote mean values ± SD. **D** Intracellular bacterial counts after 1 h of neutrophil infection. Each dot represents an experiment with neutrophils from one donor. The horizontal lines denote median values (n = 5). The level of significance was determined using Kruskal–Wallis test with Dunn’s multiple comparison post-test. **E** Assessment of SpeB positivity/negativity of indicated strains recovered from primary human neutrophils shown in **D**. Mean percentage of SpeB^+^ and SpeB^−^ clones from five independent experiments are shown (n = 5). Bacteria incubated in RPMI and THY media served as controls. **F** Distribution of SpeB^+^ and SpeB^−^ clones post neutrophil (PMN) and subsequent THY media passage. Displayed are mean [%] of five independent experiments (n = 5)

Based on our data showing the mixed SpeB phenotype particularly in the tissue milieu, we sought to identify which factors might be driving this process. Due to increased variability in SpeB phenotype of freshly isolated GAS NSTI strains, four well-characterized strains of the two dominant NSTI *emm*1 and *emm*3 types (*emm*1 strains 5448 and 8157 and *emm*3 strains 5626 and 8003) were selected. These strains displayed stable SpeB^+^ and SpeB^−^ phenotypes, respectively. All colonies of 5448, 8157 and 5626 showed a clearance zone, while colonies of 8003 did not. (Fig. 2A). Analyses of supernatants from overnight cultures showed that SpeB was readily detectable in 5448, 8157, and 5626 (Fig. 2B, Additional file 1: Fig. S8). This was further verified in a SpeB proteolytic assay (Fig. 2C). Next, the strains’ whole genome were sequenced and comparative analyses with annotated genomes of M1GAS (SF370, *emm*1) and MGAS315 (*emm*3) were performed. In general, rather low numbers of sequence variants were identified within the genomes (Additional file 1: Fig. S6). Analyses of mutational hotspots responsible for loss of SpeB expression identified a mutation in *covS* of 8003 strain resulting in T_214_P substitution (Additional file 1: Fig. S7).

Next, using the four strains with stable SpeB^+^ and SpeB^−^ phenotype, different human skin and soft tissue cell types, including keratinocytes, primary fibroblasts and immune cells (primary macrophages and neutrophils) were infected with GAS strains followed by assessment of infectivity, as well as SpeB activity by single colonies recovered from the intracellular compartment. Since clindamycin and benzylpenicillin are used to treat GAS NSTIs, control experiments with these two antibiotics were also performed (Additional file 1: Fig. S9). No differences in adherence to or internalization into keratinocytes and fibroblasts between the strains were detected (Additional file 1: Fig. S9A-B and D-E). In contrast, while the bacteria were multiplying within keratinocytes, reduced numbers of bacteria were recovered from fibroblasts over time. Only minor effects on SpeB of these two cell types were seen (Additional file 1: Fig. S9C, F). Next, primary macrophages and neutrophils were infected and intracellular bacteria were recovered. No differences in bacterial numbers were seen in macrophage infections (Additional file 1: Fig. S9G). In contrast, significantly higher numbers of SpeB^−^ 8003 strain as compared to other three SpeB^+^ stains were recovered from neutrophils (Fig. 2D). Infection of both myeloid cell types resulted in loss of SpeB activity on casein agar (Fig. 2E and Additional file 1: Fig. S9H). However, this phenotype was more pronounced in *emm*1 strains, where more than 50% of the colonies were SpeB^−^ when exposed to neutrophils (Fig. 2E). Subsequent passaging of single colonies from neutrophil experiments showed that the majority of successor generations of SpeB^−^ clones returned to a wild-type-like SpeB^+^ phenotype (Fig. 2F). Bacteria exposed to minimal inhibitory concentrations of antibiotics retained its original SpeB^+^ or SpeB^−^ phenotype (Additional file 1: Fig. S9I-K). These results demonstrate that the reversible loss of SpeB activity is seen among intracellular GAS, particularly in neutrophils, and consequently, might represent an adaptation strategy of GAS to the intracellular environment of neutrophils.

### SpeB accumulates intracellularly in GAS as a response to intracellular environment of neutrophils

To verify if the SpeB^−^ phenotype is characterized by the transient loss of SpeB expression, an impaired secretion or loss of proteolytic activity a series of experiments was performed.

**Fig. 3:**
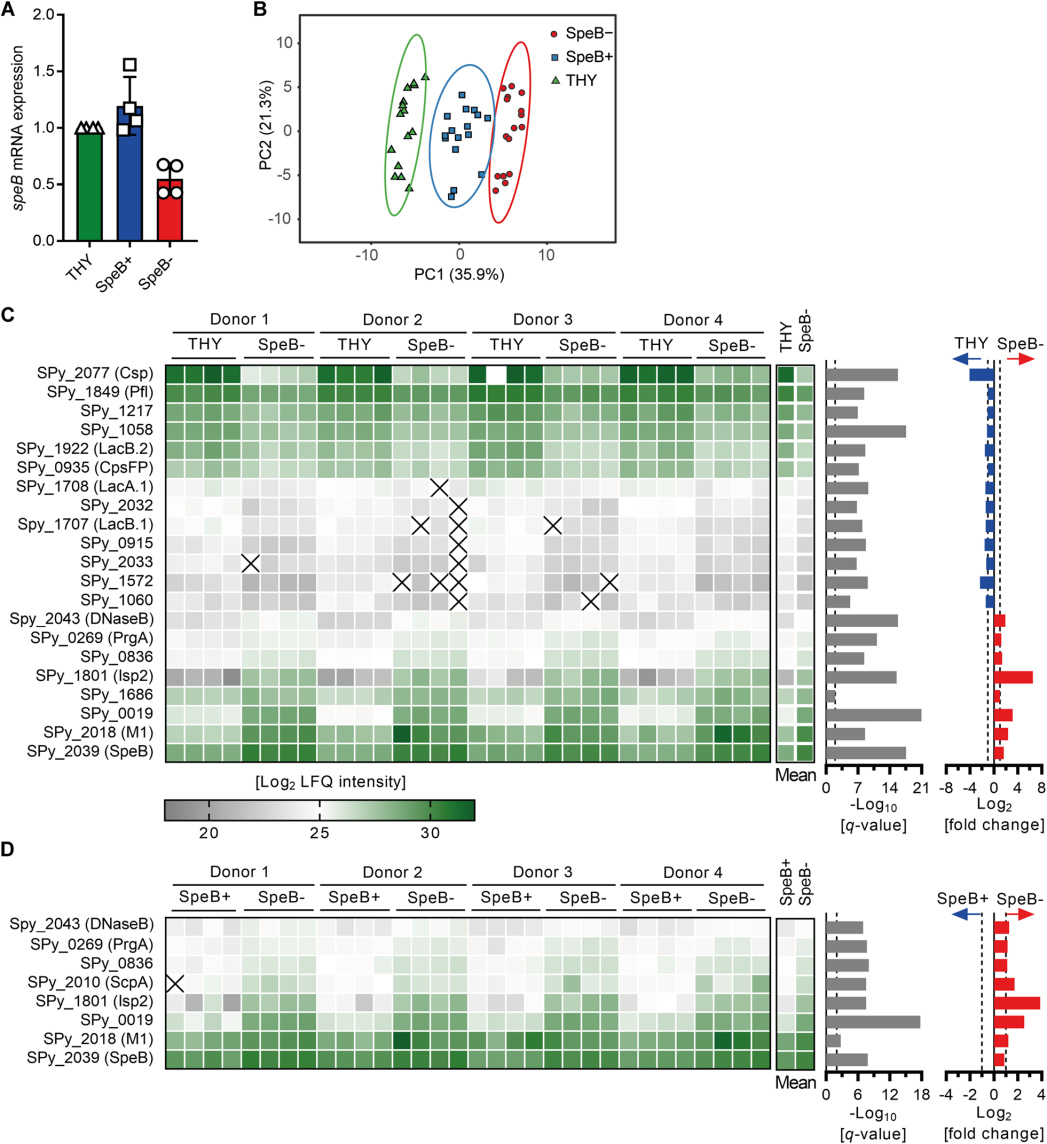
Distinct single colony proteome profile of GAS post neutrophil infection. **A** Relative *speB* mRNA expression in 5448 single colonies post neutrophil infections. Each dot represents one colony (n = 4). Bars denote mean value ± SD. **B** Principal component analysis of the proteome of single SpeB^−^ and SpeB^+^ colonies post neutrophil infections. GAS incubated in THY media, which remained 100% SpeB^+^ were used as controls. Each dot represents proteome analysis of one GAS colony (n = 16). **C, D** Heat map of differentially expressed proteins on a single colony level. Displayed are Log_2_ LFQ intensities (left panel), –Log_10_ *q*-value (middle panel), and Log_2_ fold change of the mean (right panel). Each column represents one single colony (four per condition and donor; n = 16 in total). **C** Protein abundance of THY vs. SpeB^−^ colonies and **D** SpeB^+^ vs. SpeB^−^ colonies are displayed (×, not detected)

First, relative *speB* transcription in single colonies of the 5448 strain post neutrophil infection was determined. *SpeB* transcription was detected in both SpeB^+^ as well as in SpeB^−^ colonies, albeit weaker in the latter (Fig. 3A).

Second, the proteome of SpeB^+^ and SpeB^−^ single colonies recovered from intracellular bacteria in neutrophils was quantitatively profiled by mass spectrometry. Bacteria cultured in THY media resulting in 100% SpeB^+^ served as controls. In total, up-to 752 proteins were identified in single colonies (Additional file 2: Table S3). Principal component analysis showed that all three groups (THY vs. PMN SpeB^+^ vs. PMN SpeB^−^) separate from each other, suggesting that each of them is characterized by a distinct proteome profile (Fig. 3B). Next, protein expression patterns of SpeB^−^ colonies post neutrophil infections were compared either to SpeB^+^ from the same experiment or to colonies from THY control (Fig. 3C, D). Notably, SpeB was found in all samples. In fact, SpeB^−^ GAS colonies accumulated higher amounts of SpeB intracellularly, as compared to the SpeB^+^ colonies or THY controls (Fig. 3C, D). Alignment of detected peptides with the amino acid sequence of SpeB revealed that SpeB zymogene was exclusively present intracellularly (Additional file 1: Figure S10). In addition, other secreted proteins, including DNaseB and immunogenic secreted protein 2 (Isp2) and surface anchored antiphagocytic M1 protein as well as C5a peptidase (ScpA), were found in higher abundance in SpeB^−^ colonies. Identical experiments were performed with SpeB^−^ 8003 strain. 8003 remained SpeB^−^ post neutrophil challenge and SpeB was not detected (Additional file 1: Fig. S11 and Additional file 2: Table S4).

**Fig. 4:**
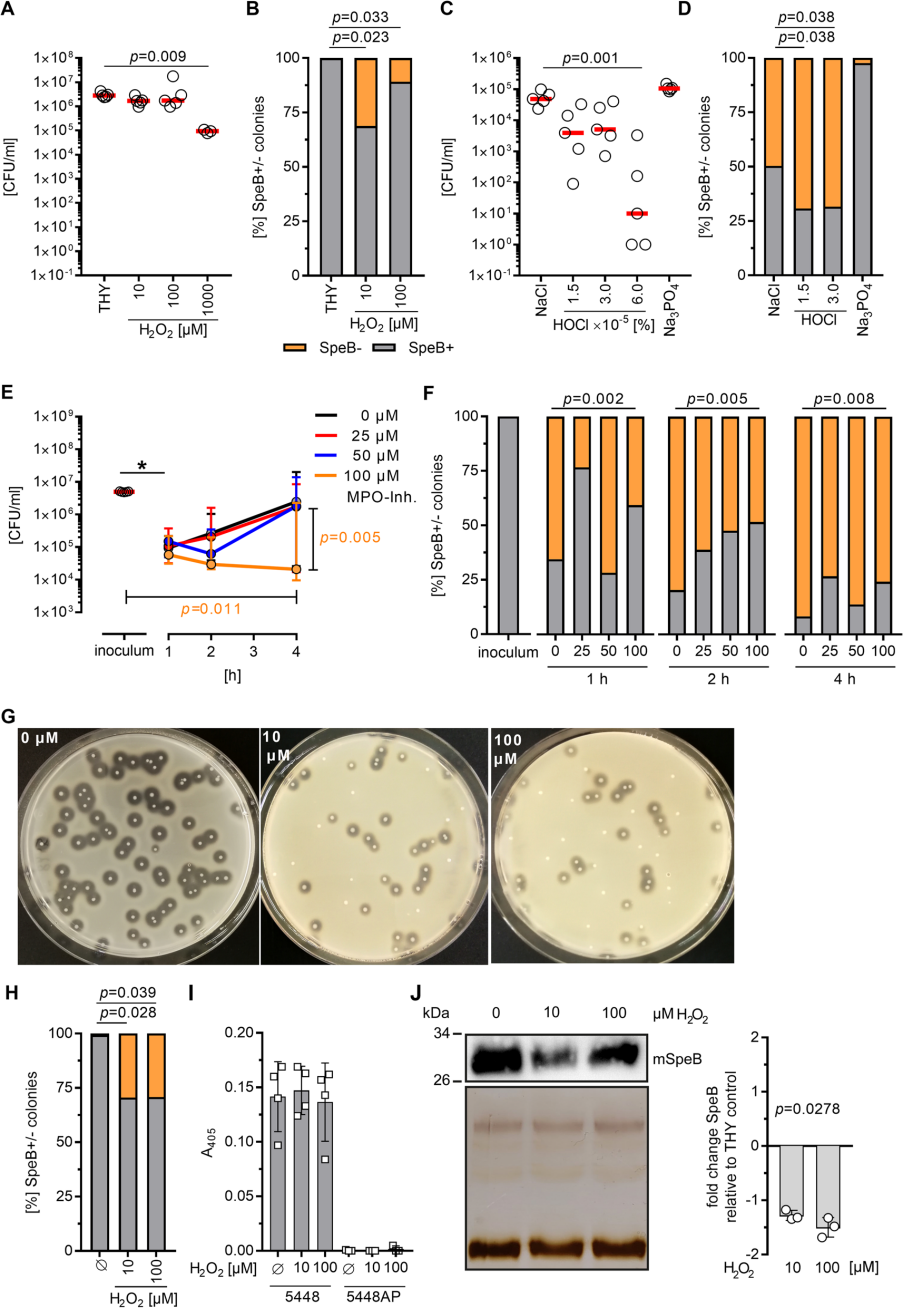
H_2_O_2_ and HOCl impair SpeB secretion by GAS. **A** GAS 5448 strain was exposed to indicated concentration of hydrogen peroxide (H_2_O_2_) and bacterial numbers were determined 1 h post stimulations. Each dot represents one independent experiment. Lines denote mean values (n = 5). **B** Assessment of SpeB positivity/negativity of 5448 strain post H_2_O_2_ treatment as displayed in **A**. Mean percentage from five independent experiments is shown (n = 5). **C** GAS 5448 strain was exposed to indicated concentration of HOCl and bacterial numbers were determined 1 h post stimulations. Each dot represents one independent experiment. Lines denote mean values (n = 5). **D** Assessment of SpeB positivity/negativity of 5448 strain post HOCl treatment as displayed in **C**. Mean percentage from five independent experiments is shown (n = 5). **A**–**D** Untreated bacteria in THY media, acidified NaCl or Na_3_PO_4_ served as controls. **E** Human primary neutrophils were infected with GAS strain 5448 and intracellular bacteria were determined by plating serial dilution of neutrophil lysates on casein agar plates post indicated time points. The MPO activity was inhibited by indicated concentrations of MPO inhibitor 30 min prior and during the entire infection period. Dots represent the median value ± range of independent experiments with five donors (n = 5). **F** Assessment of SpeB positivity/negativity of 5448 strain recovered from primary human neutrophils shown in **E**. Mean percentages of SpeB^+^ and SpeB^−^ clones from five independent experiments are shown (n = 5). **G** Representative images of GAS 5448 directly plated on casein agar containing indicated concentrations of H_2_O_2_ and **H** subsequent analyses. Mean percentage from four independent experiments is shown (n = 4). **I** SpeB activity assay (n = 4) of bacterial supernatants. GAS were grown over-night (16 h) in THY and sterile-filtered supernatants supplemented with indicated concentrations of H_2_O_2_ were used for the analyses. **J** Western blot (upper left panel), silver staining of the loading control (upper lower panel), and image analyses (right panel) of GAS 5448 supernatants post exposure to indicated concentrations of H_2_O_2_. Representative images and analysis of three independent experiments are shown (n = 3). The level of significance between the groups of experiments presented in **A–H** was determined using Kruskal Wallis test with Dunn’s posttest. The level of significance in **J** was determined using Friedman test

Third, we sought to determine which neutrophil-derived components might be responsible for the observed reversible SpeB^−^ phenotype. Therefore, an initial screen with strain 5448, which stably expresses and releases SpeB, was performed using antimicrobial peptides, lysozyme, and granule components (HBP and resistin). None of the tested agents had an impact on SpeB release/proteolytic activity by bacteria (Additional file 1: Fig. S12). Next, 5448 was exposed to different concentrations of hydrogen peroxide (H_2_O_2_). High concentrations of H_2_O_2_ (1 mM) caused a 1-log reduction in bacterial counts, while no effect on CFU were noted in the presence of 10–100 µM (Fig. 4A). Exposure of 5448 strain to these sub-lethal concentrations of H_2_O_2_ resulted in increased frequency of SpeB^−^ clones in up to 32% of the total GAS population (Fig. 4B). Since MPO converts the majority of available H_2_O_2_ intracellularly to HOCl, we next exposed strain 5448 to different concentrations of HOCl. Up to 70% of GAS did not secrete SpeB upon exposure to sub-lethal concentrations of HOCl (Fig. 4C, D). However, NaCl exposure, the source of Cl^−^, induced a similar bacterial phenotype although not to the same extent. To verify this result in a more complex setting, human primary neutrophils were infected with the 5448 strain in the presence of an MPO inhibitor and bacterial killing as well as SpeB positivity were assessed over time. To exclude potential masking effects due to the time-dependent delay in abrogated SpeB-secretion, 5448AP strain was used as a stable SpeB^−^ control. In the infection model, reduced numbers of 5448 strain were recovered from neutrophils one-hour post infection (Fig. 4E). However, increased bacterial survival was noticed over a period of the next three hours if low concentrations of MPO-inhibitor (0–50 µM) were used. Four hours post infections almost equal numbers of bacteria compared to the initial infection inoculum were recovered (Fig. 4E). High concentrations of MPO inhibitor (100 µM) reversed this effect and an increased killing of bacteria was observed over time (Fig. 4E). In congruence with those observations, the amount of SpeB^−^ clones significantly increased over the infection period. MPO-inhibition partially reversed this effect, particularly evident at 2 h post infections (Fig. 4F). In contrast, neutrophil infections with stable SpeB^−^ 5448AP strain were characterized by a continuous intracellular multiplication (Additional file 1: Fig. S13A). As expected, 5448AP remained SpeB^−^ over the entire period of infections (Fig. S13B). No MPO-inhibitor mediated effects on bacterial growth were observed (Additional file 1: Fig. S13C).

Finally, to assess whether the secretion or only the activity of SpeB were impaired by exposure of the bacteria to H_2_O_2_ within neutrophils or in liquid cultures, 5448 strain was (i) plated on casein agar containing H_2_O_2_ and (ii) SpeB presence as well as activity were determined in the presence/absence of H_2_O_2_ in bacterial supernatants. These analyses revealed that H_2_O_2_ does not impair SpeB activity but secretion in a subpopulation of the bacteria as shown via casein agar plating revealing negative colonies as well as Western blot analysis showing reduced levels of SpeB post H_2_O_2_ treatment (Fig. 4G–J; Additional file 1: Fig. S14).

### Enhanced neutrophil degranulation and presence of MPO in tissue biopsies with increased SpeB^−^ variants

**Fig. 5:**
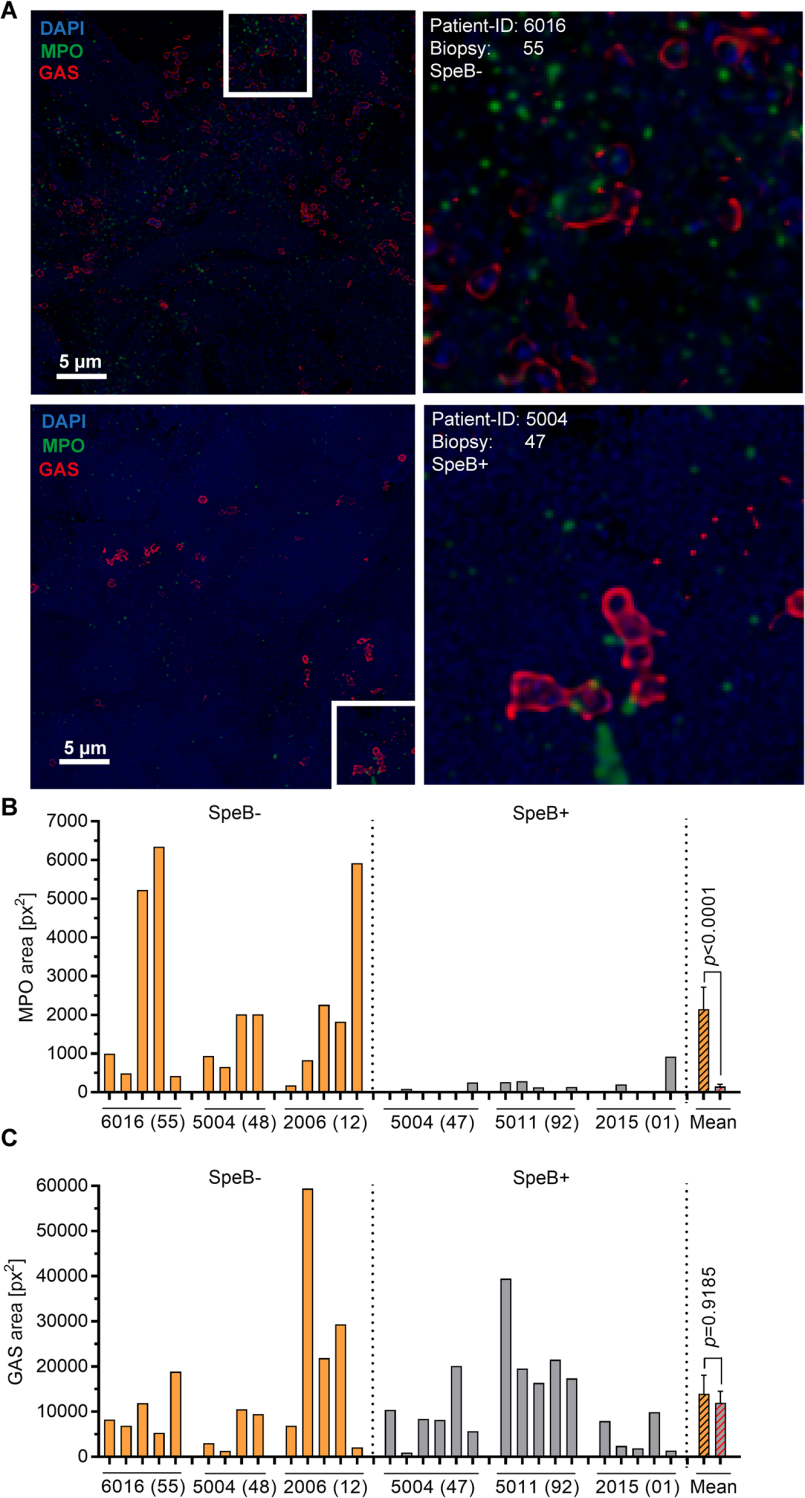
Increased levels of MPO in patient tissue biopsies associated with SpeB^−^ GAS. **A** Representative immunofluorescence micrographs of the distribution of MPO in patient biopsies are shown. **B** Image analyses of MPO and **C** GAS stainings. Calculation of the stained area (px^2^) per analyzed micrograph is presented. Each bar within diagrams denotes analysis of one image from indicated tissue biopsies. Bars on the right of each diagram denote mean values ± SD. The level of significance between the groups was determined using 2-tailed Mann–Whitney *U* test

Since MPO converts major amounts of H_2_O_2_ to HOCl, the presence of this molecule was determined in patient tissue biopsies based on the initial assessment of SpeB-positivity/negativity. Multiple tissue sections were stained for GAS and MPO, and analyzed microscopically (Fig. 5A, Additional file 1: Fig. S15). Confocal laser scanning microscopy (CLSM) and subsequent image analyses revealed that although equal bacterial load is detectable in both types of biopsies (SpeB^−^ vs. SpeB^+^), higher amounts of MPO were detected in biopsies associated with SpeB^−^ GAS clones (Fig. 5B, C). However, as evident by the micrographs, these biopsies are characterized by extensive necrosis resulting in a relatively diffuse DAPI staining (Fig. 5A). This is a characteristic of these infections and the biopsies represent tissue that are clinically indicated for surgical removal and will hence be associated with necrosis.

**Fig. 6:**
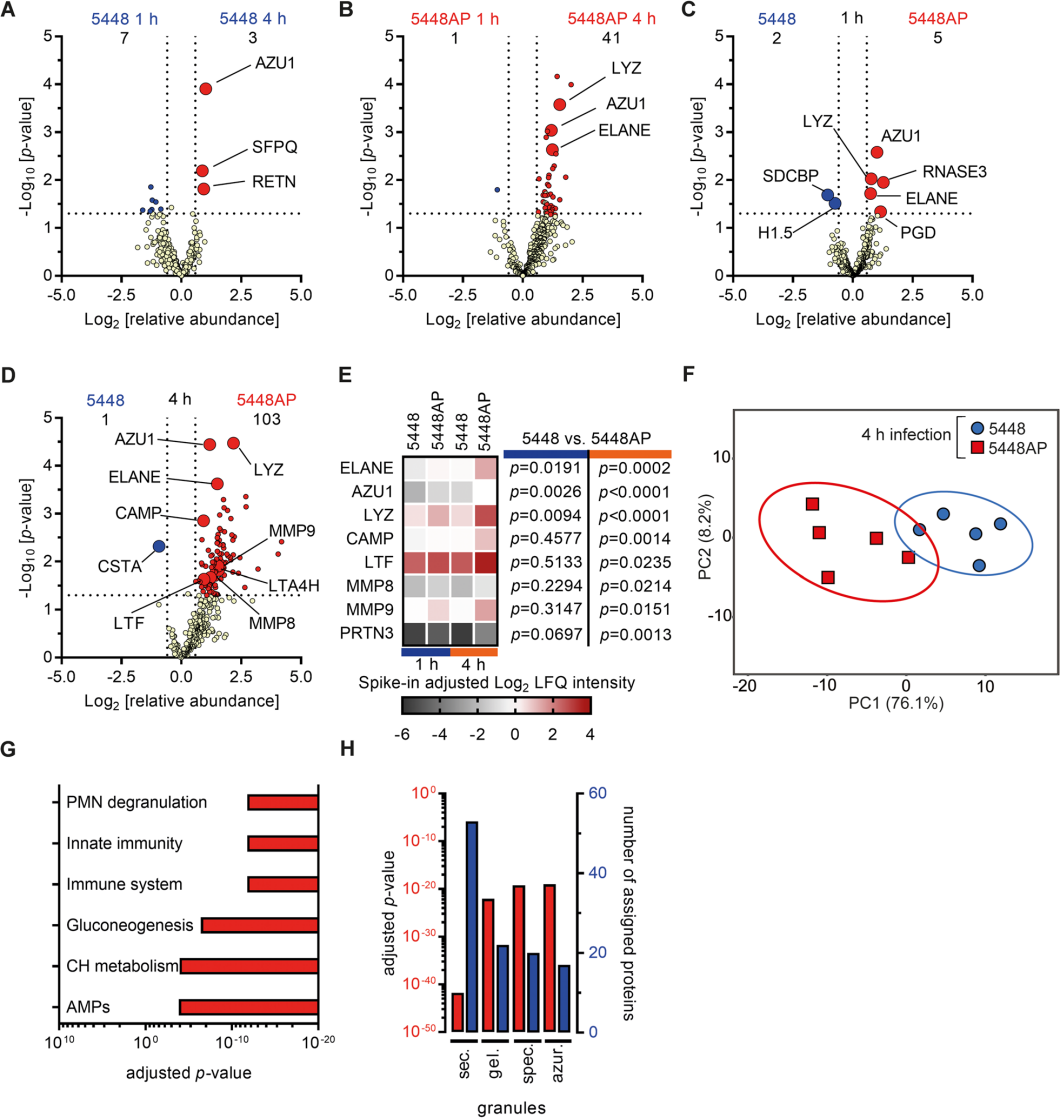
Hyper-responsiveness of human neutrophils induced by SpeB-negative GAS. **A, B** Time-dependent and **C, D** strain-dependent comparison of secretome profiles of human neutrophils exposed to 5448 or 5448AP strains. Displayed are changes in relative abundance and *p*-values of the original analyses in Additional file 2: Table S5 (n = 5). **E** Heat map highlighting some of the significant differences of protein/peptide abundance in neutrophil secretomes from (**A**–**D**). **F** Principal component analysis of secreted proteins post 4 h of infection of indicated strains. Each dot represents one donor (n = 5). The ellipses indicate the calculated 95% probability region for a bivariate normal distribution with an average center of groups. **G** Top six upregulated pathways in 5448AP infections of primary neutrophils as compared to 5448 infections. Displayed are adjusted *p-*values as determined by functional profiling in the *Reactome* database (CH, carbohydrate; AMPs, antimicrobial peptides). **H** Cellular compartment analyses of protein/peptides found in higher abundance in neutrophil secretomes of 5448AP infections as compared to 5448 infections. Displayed are adjusted *p-*values (left red axis, red bars) and number of proteins (right blue axis, blue bars). Functional profiling was performed using *Reactome* database (sec., secretory; gel., gelatinase; spec., specific; azur., azurophilic)

Both, *in vitro* as well as *ex vivo* analyses suggest that SpeB secretion is abrogated in GAS in response to H_2_O_2_ and HOCl, which subsequently triggers increased neutrophil activation resulting in enhanced tissue pathology. Therefore, we next assessed neutrophil activation on a global level. Secretome composition of neutrophils post 5448 infections was quantitatively profiled by mass spectrometry. Again, to exclude time-dependent delay in abrogated SpeB secretion of this strain, the SpeB^−^ 5448AP strain, which encodes truncated *covS* transcript effecting expression of several virulence factors (Sda1, capsule, M1-protein) [45], was used as a control. To ensure that potential alterations in secretome composition and protein abundance were not caused by differences in cytotoxicity, LDH assay was performed. Although slightly increased cytolytic events were detected over time, no differences were seen between 5448 and 5448AP infections (Additional file 1: Fig. S16). Up to 495 secreted proteins/peptides were detected in neutrophil supernatants post infections (Additional file 2: Table S5), suggesting that both, SpeB^+^ as well as SpeB^−^ strains, activate neutrophils. However, granule content release was more pronounced in infections with SpeB^−^ 5448AP strain over time (Fig. 6A, B). Comparison of neutrophil degranulation in response to 5448 vs. 5448AP strain showed that 103 proteins/peptides were more abundant in neutrophil secretome of SpeB^−^ 5448AP infections (Fig. 6C–E). Among these, several tissue degrading enzymes, including neutrophil elastase (ELANE) and metalloproteases (MMP8, MMP9) were detected. PCA revealed that neutrophil secretome of both infections is distinct at 4 h post-infections (Fig. 6F). Subsequent pathway analyses by *Reactome* profiler showed that the SpeB^−^ 5448AP strain activates several neutrophil specific pathways to a higher extent as compared to the 5448, including degranulation and immune responses (Fig. 6G, Additional file 2: Table S6). Sub-analyses of neutrophil cellular compartments showed that protein/peptides of all four granules were released in higher abundance in response to the SpeB^−^ 5448AP infections (Fig. 6H).

## Discussion

Phenotypic and genetic adaptations of GAS to the tissue environment are key survival mechanisms to establish a successful infection. In this study, we show that GAS reversibly abrogate SpeB secretion in the tissue setting resulting in improved survival of the bacteria and subsequently in higher tissue inflammation and pathology in NSTI.

Bacteria are highly adaptable species. Their genetic information and other structures are capable of alterations. Some of them are reversible when a particular pressure is lifted, whereas other alterations are maintained [7]. GAS have adapted several strategies to survive within the hostile environment in NSTIs [18, 37, 48]. One of them is the enhanced mutational rate within the *covR/S* and *ropB* genes resulting in SpeB-negative clones. Studies on these two mutational hotspots are inconclusive with respect to impact on virulence. Some studies show that these variants are less virulent, while others demonstrated that natural *covR/S* mutants are hyper-virulent [6, 45]. In humans, it was shown that SpeB is readily detectable in sera and tissues of NSTI patients [10, 16, 33] and a mixture of SpeB^+^ and SpeB^−^ GAS clones is usually seen [16, 33]. Here, we demonstrate that in addition to the irreversible loss of SpeB, due to genetic mutations, a subpopulation of GAS reversibly abrogates SpeB secretion in the tissue setting. This phenomenon is more pronounced in *emm*1 strains. Single colony proteomics showed that SpeB accumulates intracellularly and is not released. Once the tissue pressure is lifted, GAS regain the SpeB secreting function. Tissue necrosis, inflammation, neutrophil influx and degranulation are enhanced in patient biopsies associated with increased amount of SpeB^−^ GAS clones. Our data further confirm previous observations that neutrophil activation and degranulation are the main hallmarks of GAS NSTIs [14–16, 38]. These pathologic processes seem to be further intensified in biopsies with higher frequencies of the SpeB^−^ GAS. Notably, PGK, which has been demonstrated to be a potent activator of neutrophils, is highly susceptible to SpeB proteolysis and its activity can only be seen in SpeB^−^ strains [43]. Also, Zhu et al. reported that loss of SpeB resulted in increased fitness of *emm*1 GAS in a non-human primate model of necrotizing myositis [48]. Furthermore, it can be noted that in the non-human primate model neutrophil activation and degranulation was associated with tissue pathology [48].

It is widely accepted that *covR/S* mutations and the consequent total loss of SpeB result in upregulated expression of several virulence factors, which are susceptible to SpeB degradation, including Sda1, M protein, PGK, and streptolysin O. All of them facilitate pathogen’s escape from neutrophils and other immune cell types [17, 45]. Notably, GAS single colony proteomics demonstrated that in comparison to SpeB^+^, SpeB^−^ clones from the same neutrophil infections are characterized by enhanced accumulation of surface anchored M1 protein and C5a peptidase and the secreted virulence factors DNaseB and Isp2. All of these factors are of crucial importance in immune system evasion and are implicated in hyper-inflammatory processes [32, 37]. In addition, Isp was recently reported to contribute to necrotizing myositis [18]. Therefore, we speculate that transient SpeB^−^ clones might persist in the tissue for a longer period and subsequently contribute to excessive degranulation of neutrophils and hyper-inflammation. This fact is further supported by neutrophil secretome analyses. Increasing abundance of granule proteins was observed in 5448 infections over time. To rule out time-dependent effects, infections with stable hyper-virulent SpeB^−^ 5448AP strain were performed. It should be emphasized that 5448AP harbors a global regulatory mutation which affects not only SpeB secretion. This strain is further characterized by an enhanced expression of antiphagocytic M1 protein and secretion of Sda1 and Streptokinase, among others [45]. In congruence with our data, it was shown that 5448AP strain limits production of ROS, thereby ensuring it survival [46]. In contrast to the report by Williams and colleagues [46], our analyses revealed that the SpeB^−^ 5448AP strain activates neutrophils to a higher extent as compared to its parental SpeB^+^ 5448 strain. In line with this, higher abundance of resistin and MPO was detected in biopsies associated with increased amounts of SpeB^−^ GAS clones. This is in congruence with a previous report that in absence of SpeB, GAS trigger excessive neutrophil degranulation including the release of MPO [43]. MPO is associated with increased mortality in patients with sepsis [31] and a recent study demonstrated that GAS NSTIs patients had higher MPO levels in blood serum as compared to polymicrobial NSTIs. Moreover, patients presenting with STSS even significantly exceeded MPO levels of those who presented without shock [12].

Since our data suggested that neutrophils are the main cellular compartment responsible for SpeB-negativity, we subsequently analyzed the impact of neutrophil derived components on GAS phenotype. Among the tested effector molecules, H_2_O_2_ and HOCl, and Cl^−^ were responsible for the reversible loss of SpeB secretion by GAS. Neutrophils phagocytose and subsequently kill bacteria primarily through the activity of NADPH oxidase and MPO [2]. In addition, MPO can be found extracellularly through degranulation or NET formation. Both, extracellular as well as intracellular MPO converts H_2_O_2_ to HOCl through oxidation of Cl^−^ [2]. Moreover, increased survival of bacteria within neutrophils was associated with increasing percentage of SpeB^−^ clones over time. Inhibition of MPO activity resulted in significantly reduced frequency of SpeB^−^ GAS clones. However, there are certain limitations to this result: (i) NaCl, the source of Cl^−^, also impaired SpeB secretion by GAS and (ii) inhibition of MPO in neutrophils only partially reversed the SpeB^−^ phenotype. At this stage, we cannot rule out that Cl^−^ itself can induce such bacterial phenotype. Nonetheless, HOCl is a less stable compound than H_2_O_2_ and therefore, bacterial killing will only be effective in a short time. In this case, inhibition of MPO most likely resulted in H_2_O_2_ accumulation within neutrophils and in the observed improved bacterial killing.

Production of fully active SpeB is a multistage process, which includes the involvement of trigger factor (RopA), the PPIase PrsA as well as cytoplasmic SpeB inhibitor Spi (reviewed in [5]). Furthermore, GAS encode highly conserved canonical Sec pathway [9], which components are enriched at the ExPortal microdomain [30]. It was shown that cationic antimicrobial peptides, including polymyxin B, preferentially target the ExPortal. Consequently, the anionic lipids and proteins are redistributed around the peripheral membrane, which is associated with the inhibition of toxin secretion, including SpeB [44]. Whether H_2_O_2_, HOCl or Cl^−^ interfere with the above-mentioned processes remains to be elucidated.

To our knowledge, this study is the first to show that in addition to the genetically induced loss of SpeB expression, GAS transiently abrogate SpeB secretion in response to neutrophil-derived H_2_O_2_ and HOCl. SpeB^−^ clones resist phagocytic killing resulting in enhanced neutrophil degranulation, which is associated with pronounced tissue inflammation and pathology. This finding provides further insight how bacterial persistence arises in the tissue setting. Further experimental studies are warranted to determine the molecular mechanism responsible for transient loss of SpeB secretion in a subpopulation of GAS.

## Conclusions

Bacteria adapt their genetic information and phenotype to challenges they are facing during an infection. In severe GAS infections, hypervirulent SpeB-negative clones have been identified to arise due to *covR/S* and *ropB* mutations. Here we identify that such clones also arise through a transient phenotypic switch triggered by the neutrophil effector molecules hydrogen peroxide and hypochlorous acid. The resulting clones have abrogated SpeB secretion and trigger exacerbated neutrophil degranulation, which results in enhanced tissue pathology. Thus, this study identified a new virulence strategy by streptococci in severe tissue infections.

## Competing interests

The authors declare that they have no competing interests.

## Supplementary Information

**Additional file 1. **Figures S1-S16 and methods section.

**Additional file 2. **Tables S1-S6.

## Data Availability

All data associated with this study are presented in the paper and supplementary material. Whole genome sequencing data of GAS strains are available at the European Nucleotide Archive (ENA) under the reference number PRJNA 524111. The mass spectrometry proteomics data have been deposited to the ProteomeXchange Consortium via the PRIDE [29] partner repository with the dataset identifier PXD040160.

## Abbreviations

GAS: Group A streptococci
H_2_O_2_: Hydrogen peroxide
HOCl: Hypochlorous acid
MPO: Myeloperoxidase
NSTIs: Necrotizing soft tissue infections
SpeB: Streptococcal pyrogenic exotoxin B
STSS: Streptococcal toxic shock syndrome

## References

[1] Anaya DA, McMahon K, Nathens AB, Sullivan SR, Foy H, Bulger E (2005). Predictors of mortality and limb loss in necrotizing soft tissue infections. Arch Surg.

[2] Beavers WN, Skaar EP. Neutrophil-generated oxidative stress and protein damage in Staphylococcus aureus. Pathog Dis. 2016;74(6). 10.1093/femspd/ftw060 PMC5975594 27354296

[3] Bruun T, Kittang BR, de Hoog BJ, Aardal S, Flaatten HK, Langeland N, Mylvaganam H, Vindenes HA, Skrede S (2013). Necrotizing soft tissue infections caused by *Streptococcus pyogenes* and *Streptococcus dysgalactiae* subsp. equisimilis of groups C and G in western Norway. Clin Microbiol Infect.

[4] Bruun T, Rath E, Bruun Madsen M, Oppegaard O, Nekludov M, Arnell P, Karlsson Y, Babbar A, Bergey F, Itzek A, Hyldegaard O, Norrby-Teglund A, Skrede S (2020). Risk factors and predictors of mortality in streptococcal necrotizing soft-tissue infections: a multicenter prospective study. Clin Infect Dis.

[5] Carroll RK, Musser JM (2011). From transcription to activation: how group A streptococcus, the flesh-eating pathogen, regulates SpeB cysteine protease production. Mol Microbiol.

[6] Cole JN, McArthur JD, McKay FC, Sanderson-Smith ML, Cork AJ, Ranson M, Rohde M, Itzek A, Sun H, Ginsburg D, Kotb M, Nizet V, Chhatwal GS, Walker MJ (2006). Trigger for group A streptococcal M1T1 invasive disease. FASEB J.

[7] Culyba MJ, Van Tyne D (2021). Bacterial evolution during human infection: adapt and live or adapt and die. PLoS Pathog.

[8] Cuypers F, Klabunde B, Gesell Salazar M, Surabhi S, Skorka SB, Burchhardt G, Michalik S, Thiele T, Rohde M, Volker U, Hammerschmidt S, Siemens N (2020). Adenosine Triphosphate neutralizes pneumolysin-induced neutrophil activation. J Infect Dis.

[9] Ferretti JJ, McShan WM, Ajdic D, Savic DJ, Savic G, Lyon K, Primeaux C, Sezate S, Suvorov AN, Kenton S, Lai HS, Lin SP, Qian Y, Jia HG, Najar FZ, Ren Q, Zhu H, Song L, White J, Yuan X, Clifton SW, Roe BA, McLaughlin R (2001). Complete genome sequence of an M1 strain of *Streptococcus pyogenes*. Proc Natl Acad Sci U S A.

[10] Gubba S, Low DE, Musser JM (1998). Expression and characterization of group A Streptococcus extracellular cysteine protease recombinant mutant proteins and documentation of seroconversion during human invasive disease episodes. Infect Immun.

[11] Hecht A, Glasgow J, Jaschke PR, Bawazer LA, Munson MS, Cochran JR, Endy D, Salit M (2017). Measurements of translation initiation from all 64 codons in *E. coli*. Nucleic Acids Res.

[12] Hedetoft M, Bennett MH, Hyldegaard O (2021). Adjunctive hyperbaric oxygen treatment for necrotising soft-tissue infections: a systematic review and meta-analysis. Diving Hyperb Med.

[13] Hollands A, Aziz RK, Kansal R, Kotb M, Nizet V, Walker MJ (2008). A naturally occurring mutation in ropB suppresses SpeB expression and reduces M1T1 group A streptococcal systemic virulence. PLoS ONE.

[14] Johansson L, Linner A, Sunden-Cullberg J, Haggar A, Herwald H, Lore K, Treutiger CJ, Norrby-Teglund A (2009). Neutrophil-derived hyperresistinemia in severe acute streptococcal infections. J Immunol.

[15] Johansson L, Snall J, Sendi P, Linner A, Thulin P, Linder A, Treutiger CJ, Norrby-Teglund A. HMGB1 in severe soft tissue infections caused by *Streptococcus pyogenes.* Front Cell Infect Mi 2014;4. 10.3389/fcimb.2014.00004 PMC3906589 24524027

[16] Johansson L, Thulin P, Sendi P, Hertzen E, Linder A, Akesson P, Low DE, Agerberth B, Norrby-Teglund A (2008). Cathelicidin LL-37 in severe *Streptococcus pyogenes* soft tissue infections in humans. Infect Immun.

[17] Kachroo P, Eraso JM, Beres SB, Olsen RJ, Zhu L, Nasser W, Bernard PE, Cantu CC, Saavedra MO, Arredondo MJ, Strope B, Do H, Kumaraswami M, Vuopio J, Grondahl-Yli-Hannuksela K, Kristinsson KG, Gottfredsson M, Pesonen M, Pensar J, Davenport ER, Clark AG, Corander J, Caugant DA, Gaini S, Magnussen MD, Kubiak SL, Nguyen HAT, Long SW, Porter AR, DeLeo FR, Musser JM (2019). Integrated analysis of population genomics, transcriptomics and virulence provides novel insights into *Streptococcus pyogenes* pathogenesis. Nat Genet.

[18] Kachroo P, Eraso JM, Olsen RJ, Zhu L, Kubiak SL, Pruitt L, Yerramilli P, Cantu CC, Ojeda Saavedra M, Pensar J, Corander J, Jenkins L, Kao L, Granillo A, Porter AR, DeLeo FR, Musser JM. New pathogenesis mechanisms and translational leads identified by multidimensional analysis of necrotizing myositis in primates. mBio 2020;11(1). 10.1128/mBio.03363-19 PMC7029145 32071274

[19] Kaul R, McGeer A, Low DE, Green K, Schwartz B (1997). Population-based surveillance for group A streptococcal necrotizing fasciitis: clinical features, prognostic indicators, and microbiologic analysis of seventy-seven cases. Ontario Group A Streptococcal Study. Am J Med.

[20] Kubica M, Guzik K, Koziel J, Zarebski M, Richter W, Gajkowska B, Golda A, Maciaq-Gudowska A, Brix K, Shaw L, Foster T, Potempa J (2008). A potential new pathway for *Staphylococcus aureus* dissemination: the silent survival of S aureus phagocytosed by human monocyte-derived macrophages. PLoS ONE.

[21] Ly AT, Noto JP, Walwyn OL, Tanz RR, Shulman ST, Kabat W, Bessen DE (2017). Differences in SpeB protease activity among group A streptococci associated with superficial, invasive, and autoimmune disease. PLoS ONE.

[22] Madsen MB, Skrede S, Perner A, Arnell P, Nekludov M, Bruun T, Karlsson Y, Hansen MB, Polzik P, Hedetoft M, Rosen A, Saccenti E, Bergey F, MartinsDos Santos VAP, Norrby-Teglund A, Hyldegaard O (2019). Patient’s characteristics and outcomes in necrotising soft-tissue infections: results from a Scandinavian, multicentre, prospective cohort study. Intensive Care Med.

[23] Mairpady SS, Siemens N, Monk IR, Mohan DB, Mukundan S, Krishnan KC, Prabhakara S, Snall J, Kearns A, Vandenesch F, Svensson M, Kotb M, Gopal B, Arakere G, Norrby-Teglund A (2016). A point mutation in AgrC determines cytotoxic or colonizing properties associated with phenotypic variants of ST22 MRSA strains. Sci Rep.

[24] Nauseef WM (2014). Myeloperoxidase in human neutrophil host defence. Cell Microbiol.

[25] Nelson DC, Garbe J, Collin M (2011). Cysteine proteinase SpeB from *Streptococcus pyogenes*—a potent modifier of immunologically important host and bacterial proteins. Biol Chem.

[26] Norrby-Teglund A, Thulin P, Gan BS, Kotb M, McGeer A, Andersson J, Low DE (2001). Evidence for superantigen involvement in severe group a streptococcal tissue infections. J Infect Dis.

[27] Palma Medina LM, Rath E, Jahagirdar S, Bruun T, Madsen MB, Stralin K, Unge C, Hansen MB, Arnell P, Nekludov M, Hyldegaard O, Lourda M, Santos V, Saccenti E, Skrede S, Svensson M, Norrby-Teglund A. Discriminatory plasma biomarkers predict specific clinical phenotypes of necrotizing soft-tissue infections. J Clin Invest. 2021;131(14). 10.1172/JCI149523 PMC8279592 34263738

[28] Papayannopoulos V, Metzler KD, Hakkim A, Zychlinsky A (2010). Neutrophil elastase and myeloperoxidase regulate the formation of neutrophil extracellular traps. J Cell Biol.

[29] Perez-Riverol Y, Bai J, Bandla C, Garcia-Seisdedos D, Hewapathirana S, Kamatchinathan S, Kundu DJ, Prakash A, Frericks-Zipper A, Eisenacher M, Walzer M, Wang S, Brazma A, Vizcaino JA (2022). The PRIDE database resources in 2022: a hub for mass spectrometry-based proteomics evidences. Nucleic Acids Res.

[30] Rosch JW, Caparon MG (2005). The ExPortal: an organelle dedicated to the biogenesis of secreted proteins in *Streptococcus pyogenes*. Mol Microbiol.

[31] Schrijver IT, Kemperman H, Roest M, Kesecioglu J, de Lange DW (2017). Myeloperoxidase can differentiate between sepsis and non-infectious SIRS and predicts mortality in intensive care patients with SIRS. Intensive Care Med Exp.

[32] Shumba P, Mairpady Shambat S, Siemens N (2019). The role of streptococcal and staphylococcal exotoxins and proteases in human necrotizing soft tissue infections. Toxins (Basel).

[33] Siemens N, Chakrakodi B, Shambat SM, Morgan M, Bergsten H, Hyldegaard O, Skrede S, Arnell P, Madsen MB, Johansson L, Juarez J, Bosnjak L, Morgelin M, Svensson M, Norrby-Teglund A (2016). Biofilm in group A streptococcal necrotizing soft tissue infections. JCI Insight.

[34] Siemens N, Fiedler T, Normann J, Klein J, Munch R, Patenge N, Kreikemeyer B (2012). Effects of the ERES pathogenicity region regulator Ralp3 on Streptococcus pyogenes serotype M49 virulence factor expression. J Bacteriol.

[35] Siemens N, Kittang BR, Chakrakodi B, Oppegaard O, Johansson L, Bruun T, Mylvaganam H, Svensson M, Skrede S, Norrby-Teglund A (2015). Increased cytotoxicity and streptolysin O activity in group G streptococcal strains causing invasive tissue infections. Sci Rep.

[36] Siemens N, Oehmcke-Hecht S, Hossmann J, Skorka SB, Nijhuis RHT, Ruppen C, Skrede S, Rohde M, Schultz D, Lalk M, Itzek A, Pieper DH, van den Bout CJ, Claas ECJ, Kuijper EJ, Mauritz R, Sendi P, Wunderink HF, Norrby-Teglund A (2019). Prothrombotic and proinflammatory activities of the beta-hemolytic group b streptococcal pigment. J Innate Immun.

[37] Siemens N, Snall J, Svensson M, Norrby-Teglund A (2020). Pathogenic mechanisms of streptococcal necrotizing soft tissue infections. Adv Exp Med Biol.

[38] Snall J, Linner A, Uhlmann J, Siemens N, Ibold H, Janos M, Linder A, Kreikemeyer B, Herwald H, Johansson L, Norrby-Teglund A (2016). Differential neutrophil responses to bacterial stimuli: streptococcal strains are potent inducers of heparin-binding protein and resistin-release. Sci Rep.

[39] Stevens DL, Bryant AE (2017). Necrotizing soft-tissue infections. N Engl J Med.

[40] Sumby P, Whitney AR, Graviss EA, DeLeo FR, Musser JM (2006). Genome-wide analysis of group a streptococci reveals a mutation that modulates global phenotype and disease specificity. PLoS Pathog.

[41] Thanert R, Itzek A, Hossmann J, Hamisch D, Madsen MB, Hyldegaard O, Skrede S, Bruun T, Norrby-Teglund A, Medina E, Pieper DH (2019). Molecular profiling of tissue biopsies reveals unique signatures associated with streptococcal necrotizing soft tissue infections. Nat Commun.

[42] Thulin P, Johansson L, Low DE, Gan BS, Kotb M, McGeer A, Norrby-Teglund A (2006). Viable group A streptococci in macrophages during acute soft tissue infection. PLoS Med.

[43] Uhlmann J, Siemens N, Kai-Larsen Y, Fiedler T, Bergman P, Johansson L, Norrby-Teglund A (2016). Phosphoglycerate kinase-a novel streptococcal factor involved in neutrophil activation and degranulation. J Infect Dis.

[44] Vega LA, Caparon MG (2012). Cationic antimicrobial peptides disrupt the *Streptococcus pyogenes* ExPortal. Mol Microbiol.

[45] Walker MJ, Hollands A, Sanderson-Smith ML, Cole JN, Kirk JK, Henningham A, McArthur JD, Dinkla K, Aziz RK, Kansal RG, Simpson AJ, Buchanan JT, Chhatwal GS, Kotb M, Nizet V (2007). DNase Sda1 provides selection pressure for a switch to invasive group A streptococcal infection. Nat Med.

[46] Williams JG, Ly D, Geraghty NJ, McArthur JD, Vyas HKN, Gorman J, Tsatsaronis JA, Sluyter R, Sanderson-Smith ML (2020). *Streptococcus pyogenes* M1T1 variants induce an inflammatory neutrophil phenotype including activation of inflammatory caspases. Front Cell Infect Microbiol.

[47] Witko-Sarsat V, Rieu P, Descamps-Latscha B, Lesavre P, Halbwachs-Mecarelli L (2000). Neutrophils: molecules, functions and pathophysiological aspects. Lab Invest.

[48] Zhu L, Olsen RJ, Beres SB, Eraso JM, Saavedra MO, Kubiak SL, Cantu CC, Jenkins L, Charbonneau ARL, Waller AS, Musser JM (2019). Gene fitness landscape of group A streptococcus during necrotizing myositis. J Clin Invest.

