# Supplementary material for: Neutrophil-derived reactive agents induce a transient SpeB negative phenotype in Streptococcus pyogenes
Source: J Biomed Sci. 2023 Jul 10;30:52. doi: 10.1186/s12929-023-00947-x (PMC10331992; doi:10.1186/s12929-023-00947-x)
Supplement: Supplementary file 1 — Additional file 1. Figures S1-S16 and methods section. [file 12929_2023_947_MOESM1_ESM.pdf]

Supplemental Material

Neutrophil-derived reactive agents induce a transient SpeB negative phenotype in *Streptococcus pyogenes*

Patience Shumba[1], Thomas Sura[2], Kirsten Moll[3], Bhavya Chakrakodi[3], Lea A. Tölken[1], Jörn Hoßmann[4], Katharina J. Hoff[5], Ole Hyldegaard[6,7], Michael Nekludov[8], Mattias Svensson[3], Per Arnell[9], Steinar Skrede[10,11], INFECT Study Group[12], Anna Norrby-Teglund[3#], and Nikolai Siemens[1#*]

[1]Department of Molecular Genetics and Infection Biology, University of Greifswald, Greifswald, Germany

[2]Department of Microbial Proteomics, Institute of Microbiology, University of Greifswald, Greifswald, Germany

[3]Center for Infectious Medicine, Karolinska Institutet, Karolinska University Hospital, Huddinge, Stockholm, Sweden

[4]Helmholtz Center for Infection Research, Braunschweig, Germany

[5]Institute of Mathematics and Computer Science, University of Greifswald, Greifswald, Germany

[6]Department of Anaesthesia, Head and Orthopedic Center, University Hospital Copenhagen, Rigshospitalet, Copenhagen, Denmark

[7]Institute of Clinical Medicine, University of Copenhagen, Copenhagen, Denmark

[8]Department of Anaesthesia, Surgical Services and Intensive Care, Karolinska Institute, Karolinska University Hospital, Stockholm, Sweden

[9]Department of Anaesthesiology and Intensive Care Medicine, Sahlgrenska University Hospital, Gothenburg, Sweden

[10]Department of Medicine, Haukeland University Hospital, Bergen, Norway

[11]Department of Clinical Science, University of Bergen, Bergen, Norway

[12]INFECT Study Group (Morten Hedetoft[6], Trond Bruun, Oddvar Oppegaard[10,11], Torbjørn Nedrebø[13], Eivind Rath[10], Martin Bruun Madsen[14])

[13]Department of Anaesthesia and Intensive care, Haukeland University Hospital, Bergen, Norway

[14]Department of Intensive Care, Rigshospitalet, University of Copenhagen, Copenhagen, Denmark

[#]ANT and NS contributed equally to this work

***Correspondance:** Nikolai Siemens; **Email:** nikolai.siemens@uni-greifswald.de

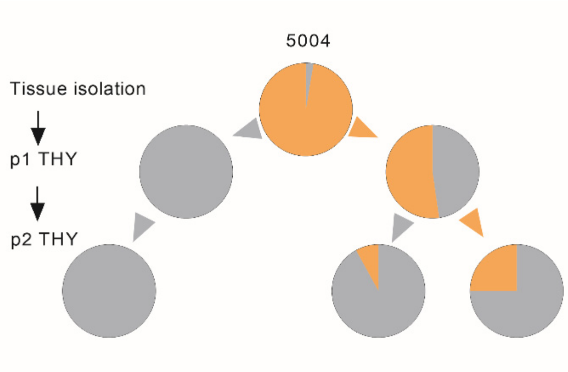

Fig. S1. Reversible loss of SpeB secretion after tissue recovery. Sections of the tissue biopsy of patient 5004 were cultured on casein agar plates.SpeB+ and SpeB- colonies were recovered and passaged in THY media over night. SpeB-positivity/-negativity were assessed via casein agar assay (SpeB+ [grey]; SpeB- [orange]).

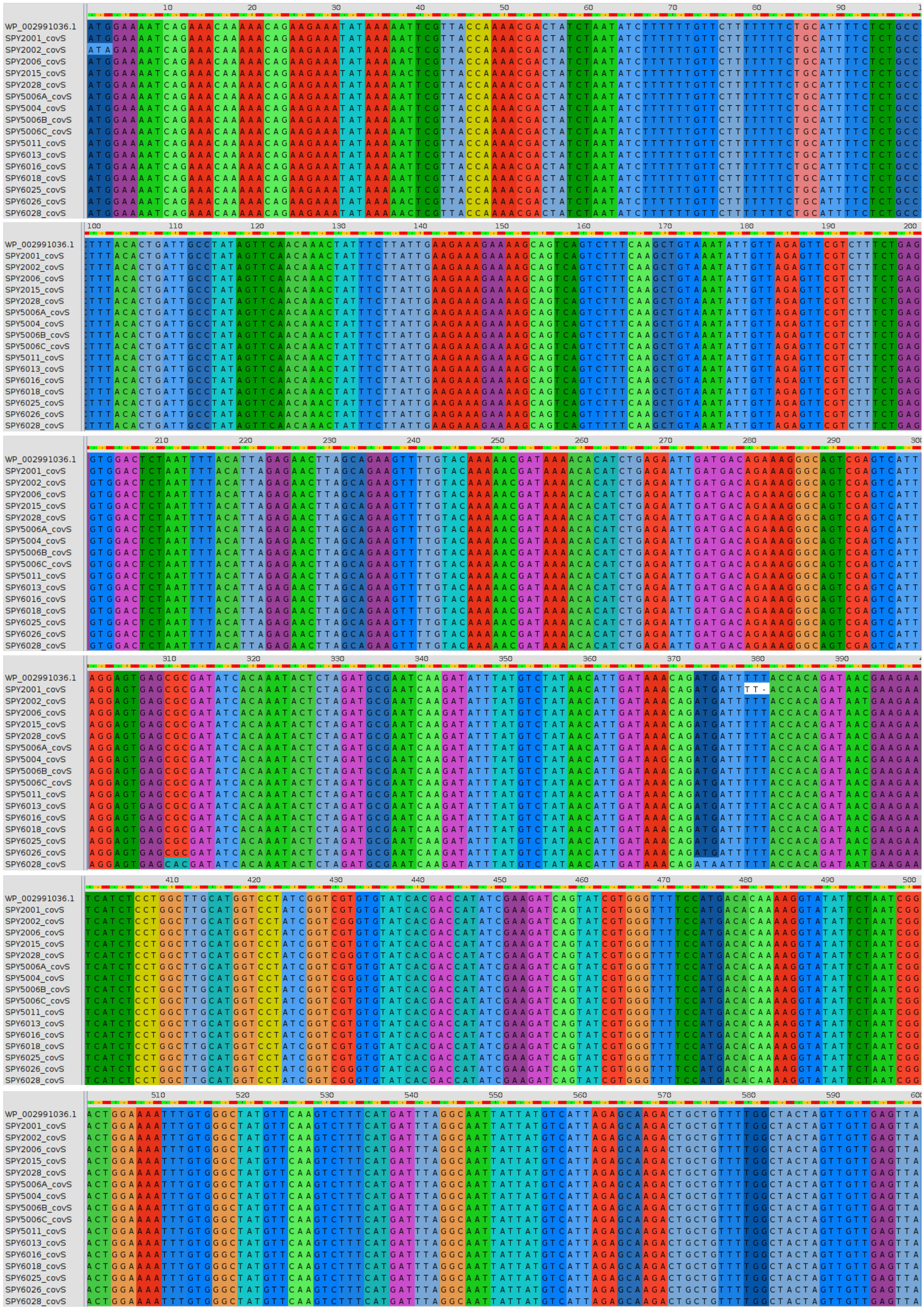
WP_002991036.1
SPY2001_covS
SPY2002_covS
SPY2006_covS
SPY2015_covS
SPY2028_covS
SPY5006A_covS
SPY5004_covS
SPY5006B_covS
SPY5006C_covS
SPY5011_covS
SPY6013_covS
SPY6016_covS
SPY6018_covS
SPY6025_covS
SPY6026_covS
SPY6028_covS

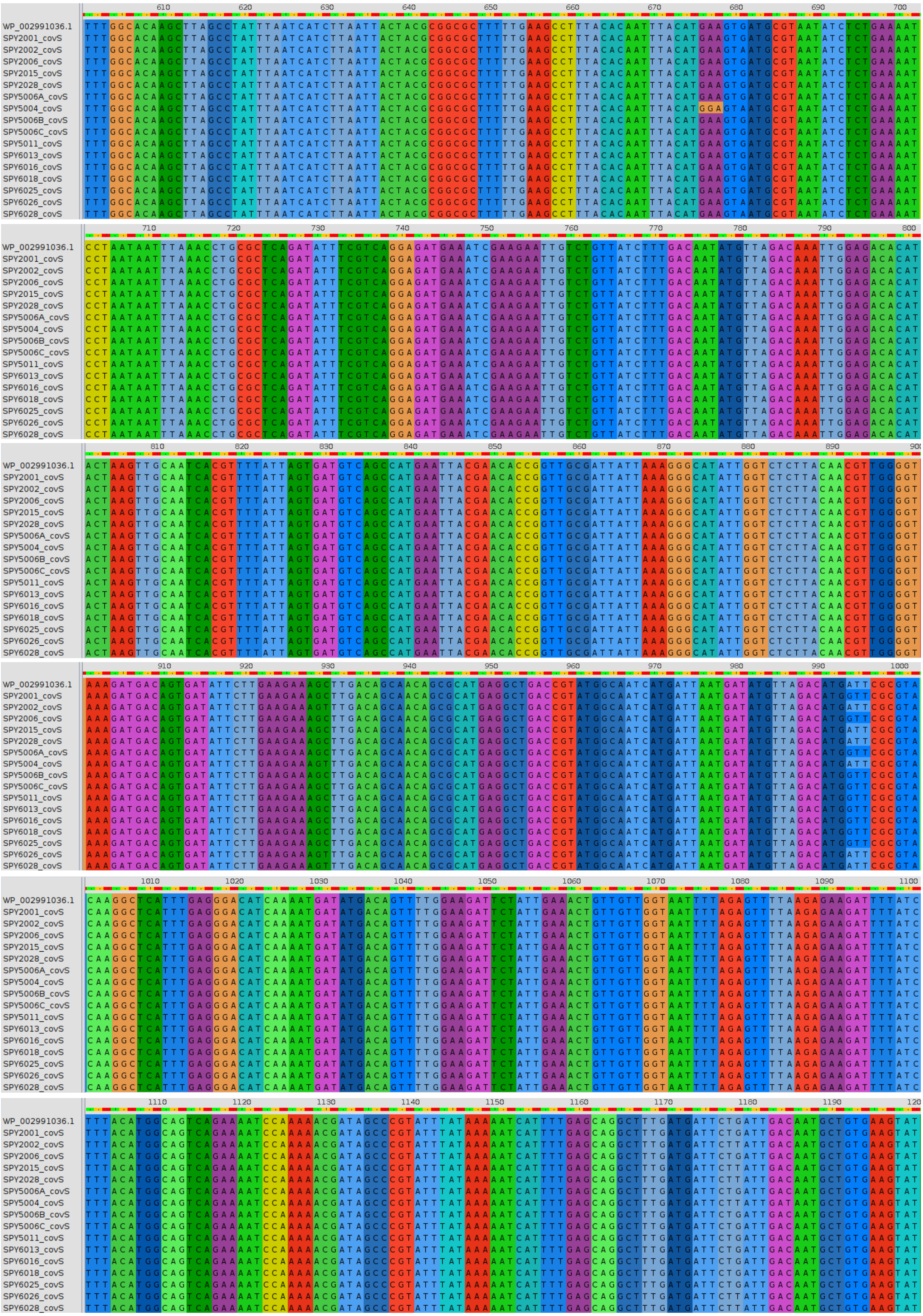
610 620 630 640 650 660 670 680 690 700
710 720 730 740 750 760 770 780 790 800
810 820 830 840 850 860 870 880 890 900
910 920 930 940 950 960 970 980 990 1000
1010 1020 1030 1040 1050 1060 1070 1080 1090 1100
1110 1120 1130 1140 1150 1160 1170 1180 1190 1200
WP_002991036.1
SPY2001_covS
SPY2002_covS
SPY2006_covS
SPY2015_covS
SPY2028_covS
SPY5006A_covS
SPY5004_covS
SPY5006B_covS
SPY5006C_covS
SPY5011_covS
SPY6013_covS
SPY6016_covS
SPY6018_covS
SPY6025_covS
SPY6026_covS
SPY6028_covS

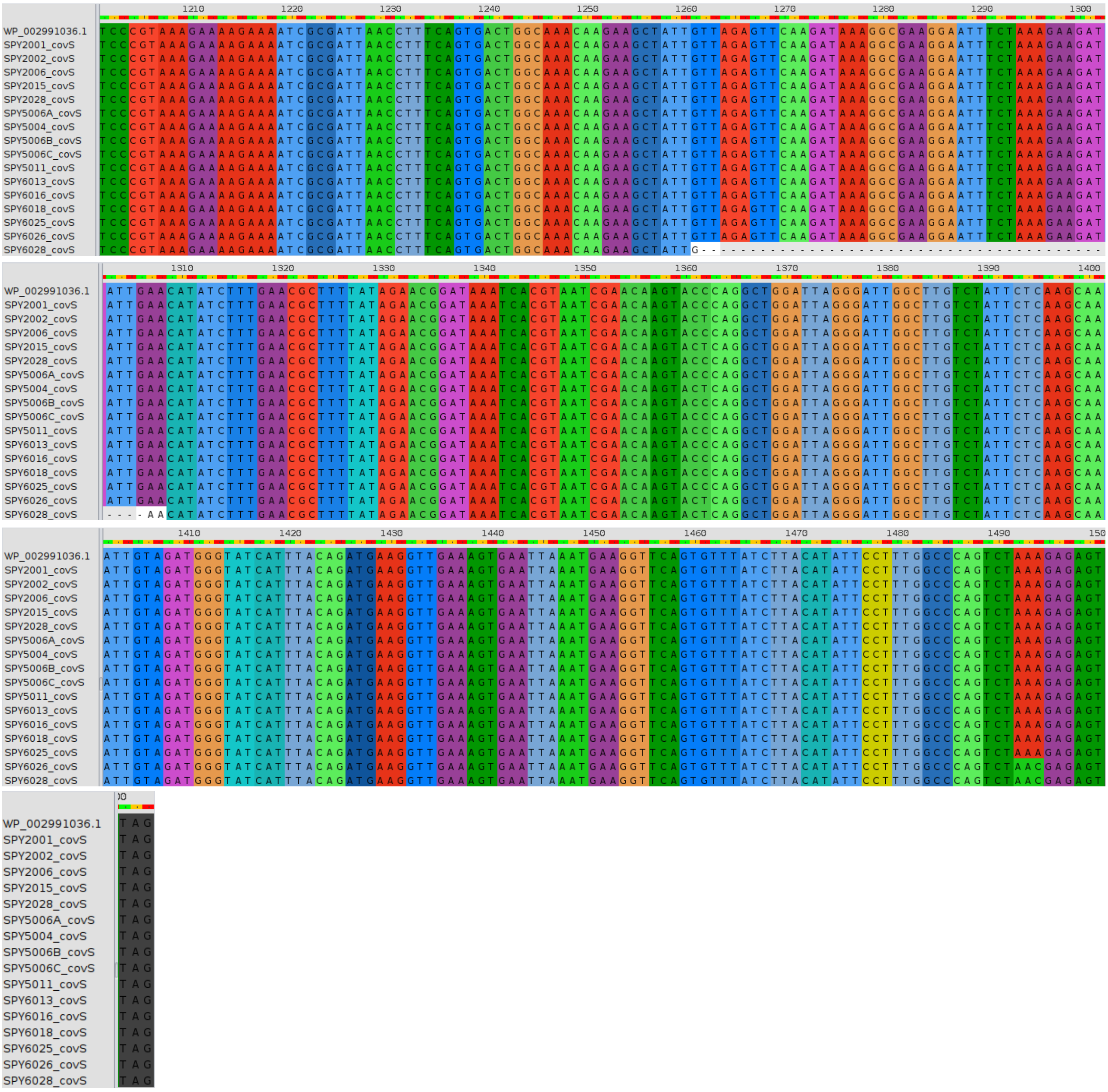

Fig. S2. ***CovS*** **gene sequence alignment of indicated strains isolated from NSTI patient´s biopsies** (Figure 1A).

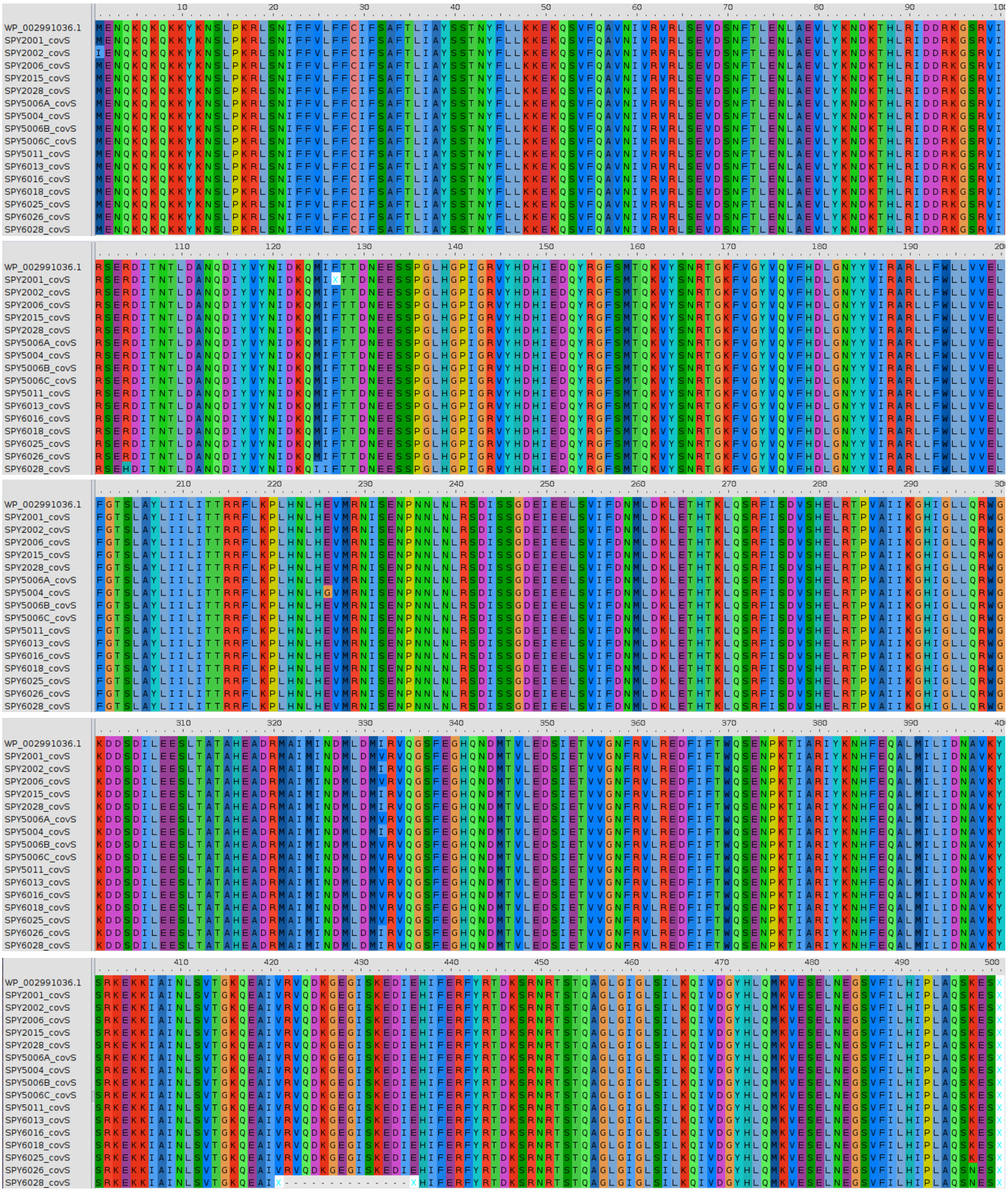

Fig. S3. CovS amino acid sequence alignment of indicated strains isolated from NSTI patient´s biopsies (Figure 1A).

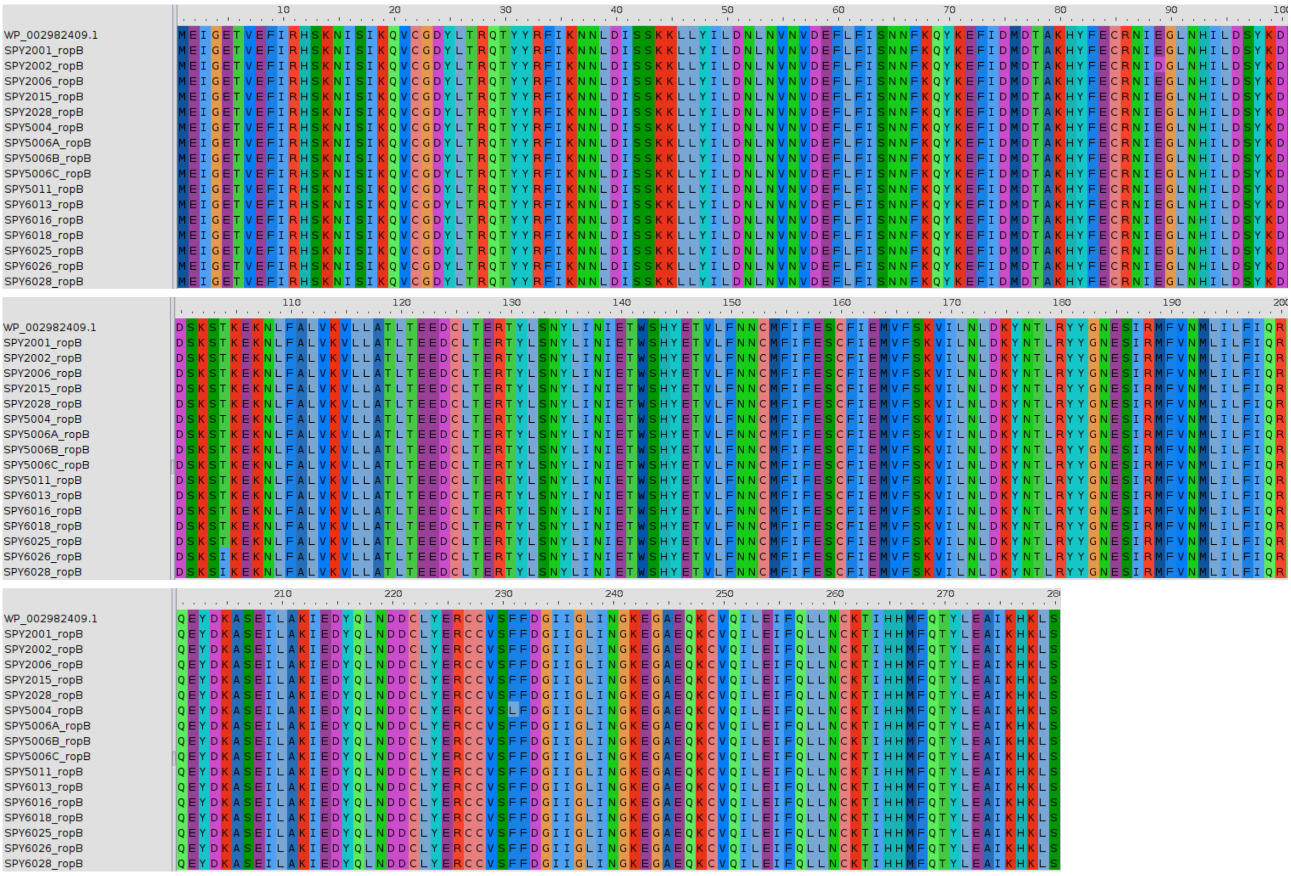

Fig. S4. RopB amino acid sequence alignment of indicated strains isolated from NSTI patient´s biopsies (Figure 1A).

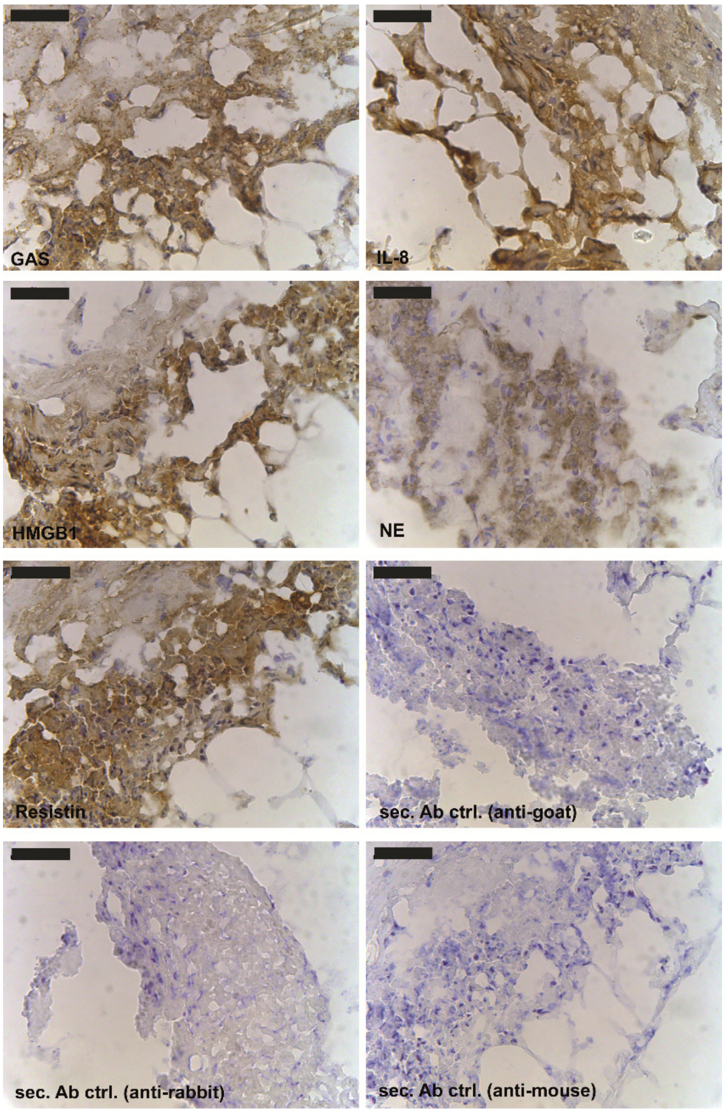

Fig. S5. Inflammation and phagocytic infiltration at the local site of infection. Representative immuno-histochemically stained tissue biopsies from GAS NSTI patients. (GAS, group A streptococcus; HMGB1, High-mobility group protein B1; NE, neutrophil elastase; black bars: 50 µm).

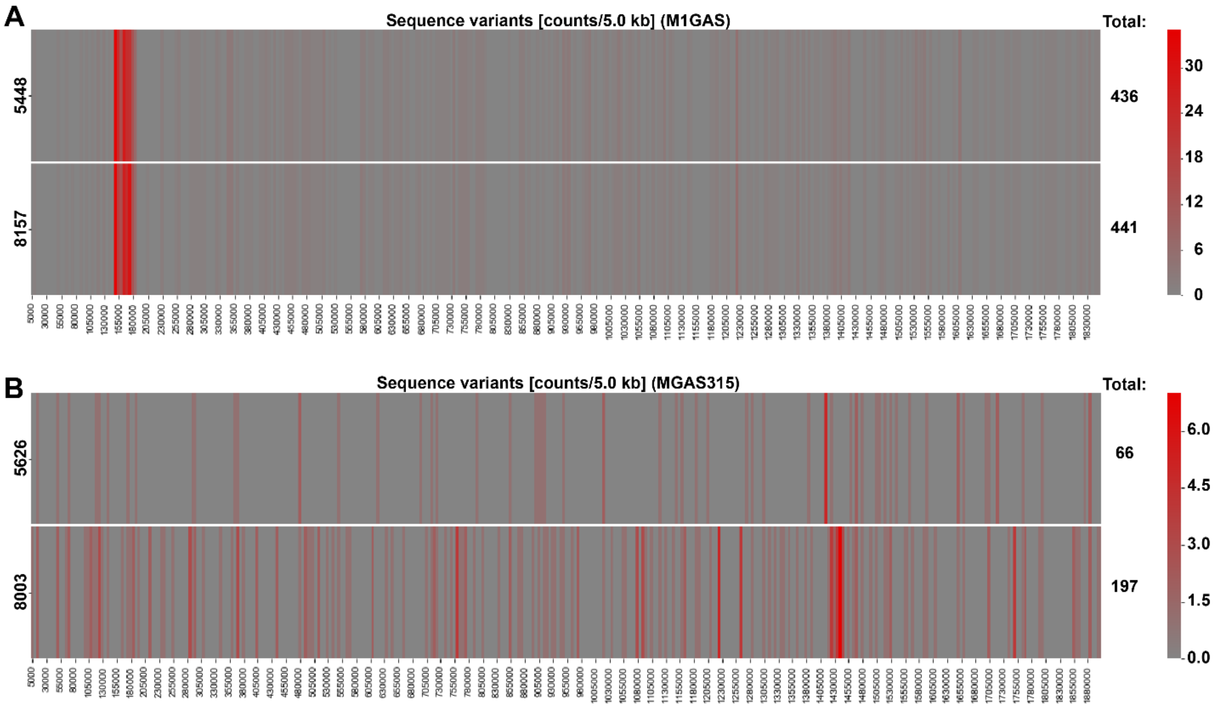

Figure S6. Whole genome sequencing analyses. Base differences per 5.0 kb based on the comparison of the whole genomes of the indicated strains with the annotated genomes of (**A**) M1GAS (*emm1* strains) and (**B**) MGAS315 (*emm3* strains).

```
SPy_0336          MENQKQKQKKYKNSLPKRLSNIFFVLFFCIFSAFTLIAYSSTNYFLLKKEKQSVFQAVNI
SpyM3_0245        MENQKQKQKKYKNSLPKRLSNIFFVLFFCIFSAFTLIAYSSTNYFLLKKEKQSVFQAVNI
CNGIIICF_01748    MENQKQKQKKYKNSLPKRLSNIFFVLFFCIFSAFTLIAYSSTNYFLLKKEKQSVFQAVNI
IKGFMBIA_01747    MENQKQKQKKYKNSLPKRLSNIFFVLFFCIFSAFTLIAYSSTNYFLLKKEKQSVFQAVNI
DNELCKEF_00023    MENQKQKQKKYKNSLPKRLSNIFFVLFFCIFSAFTLIAYSSTNYFLLKKEKQSVFQAVNI
LCNJLCPO_00752    MENQKQKQKKYKNSLPKRLSNIFFVLFFCIFSAFTLIAYSSTNYFLLKKEKQSVFQAVNI
                  ************************************************************

SPy_0336          VRVRLSEVDSNFTLENLAEVLYKNDKTHLRIDDRKGSRVIRSERDITNTLDANQDIYVYN
SpyM3_0245        VRVRLSEVDSNFTLENLAEVLYKNDKTHLRIDDRKGSRVIRSERDITNTLDANQDIYVYN
CNGIIICF_01748    VRVRLSEVDSNFTLENLAEVLYKNDKTHLRIDDRKGSRVIRSERDITNTLDANQDIYVYN
IKGFMBIA_01747    VRVRLSEVDSNFTLENLAEVLYKNDKTHLRIDDRKGSRVIRSERDITNTLDANQDIYVYN
DNELCKEF_00023    VRVRLSEVDSNFTLENLAEVLYKNDKTHLRIDDRKGSRVIRSERDITNTLDANQDIYVYN
LCNJLCPO_00752    VRVRLSEVDSNFTLENLAEVLYKNDKTHLRIDDRKGSRVIRSERDITNTLDANQDIYVYN
                  ************************************************************

SPy_0336          IDKQMIFTTDNEESSPGLHGPIGRVYHDHIEDQYRGFSMTQKVYSNRTGKFVGYVQVFHD
SpyM3_0245        IDKQMIFTTDNEESSPGLHGPIGRVYHDHIEDQYRGFSMTQKVYSNRTGKFVGYVQVFHD
CNGIIICF_01748    IDKQMIFTTDNEESSPGLHGPIGRVYHDHIEDQYRGFSMTQKVYSNRTGKFVGYVQVFHD
IKGFMBIA_01747    IDKQMIFTTDNEESSPGLHGPIGRVYHDHIEDQYRGFSMTQKVYSNRTGKFVGYVQVFHD
DNELCKEF_00023    IDKQMIFTTDNEESSPGLHGPIGRVYHDHIEDQYRGFSMTQKVYSNRTGKFVGYVQVFHD
LCNJLCPO_00752    IDKQMIFTTDNEESSPGLHGPIGRVYHDHIEDQYRGFSMTQKVYSNRTGKFVGYVQVFHD
                  ************************************************************

SPy_0336          LGNYYVIRARLLFWLLVVELFGTSLAYLIILITTRRFLKPLHNLHEVMRNISENPNNLNL
SpyM3_0245        LGNYYVIRARLLFWLLVVELFGTSLAYLIILITTRRFLKPLHNLHEVMRNISENPNNLNL
CNGIIICF_01748    LGNYYVIRARLLFWLLVVELFGTSLAYLIILITTRRFLKPLHNLHEVMRNISENPNNLNL
IKGFMBIA_01747    LGNYYVIRARLLFWLLVVELFGTSLAYLIILITTRRFLKPLHNLHEVMRNISENPNNLNL
DNELCKEF_00023    LGNYYVIRARLLFWLLVVELFGTSLAYLIILITTRRFLKPLHNLHEVMRNISENPNNLNL
LCNJLCPO_00752    LGNYYVIRARLLFWLLVVELFGTSLAYLIILITPRRFLKPLHNLHEVMRNISENPNNLNL
                  ********************************* **************************

SPy_0336          RSDISSGDEIEELSVIFDNMLDKLETHTKLQSRFISDVSHELRTPVAIIKGHIGLLQRWG
SpyM3_0245        RSDISSGDEIEELSVIFDNMLDKLETHTKLQSRFISDVSHELRTPVAIIKGHIGLLQRWG
CNGIIICF_01748    RSDISSGDEIEELSVIFDNMLDKLETHTKLQSRFISDVSHELRTPVAIIKGHIGLLQRWG
IKGFMBIA_01747    RSDISSGDEIEELSVIFDNMLDKLETHTKLQSRFISDVSHELRTPVAIIKGHIGLLQRWG
DNELCKEF_00023    RSDISSGDEIEELSVIFDNMLDKLETHTKLQSRFISDVSHELRTPVAIIKGHIGLLQRWG
LCNJLCPO_00752    RSDISSGDEIEELSVIFDNMLDKLETHTKLQSRFISDVSHELRTPVAIIKGHIGLLQRWG
                  ************************************************************

SPy_0336          KDDSDILEESLTATAHEADRMAIMINDMLDMIRVQGSFEGHQNDMTVLEDSIETVVGNFR
SpyM3_0245        KDDSDILEESLTATAHEADRMAIMINDMLDMIRVQGSFEGHQNDMTVLEDSIETVVGNFR
CNGIIICF_01748    KDDSDILEESLTATAHEADRMAIMINDMLDMVRVQGSFEGHQNDMTVLEDSIETVVGNFR
IKGFMBIA_01747    KDDSDILEESLTATAHEADRMAIMINDMLDMVRVQGSFEGHQNDMTVLEDSIETVVGNFR
DNELCKEF_00023    KDDSDILEESLTATAHEADRMAIMINDMLDMIRVQGSFEGHQNDMTVLEDSIETVVGNFR
LCNJLCPO_00752    KDDSDILEESLTATAHEADRMAIMINDMLDMIRVQGSFEGHQNDMTVLEDSIETVVGNFR
                  *******************************:****************************

SPy_0336          VLREDFIFTWQSENPKTIARIYKNHFEQALMILIDNAVKYSRKEKKIAINLSVTGKQEAI
SpyM3_0245        VLREDFIFTWQSENPKTIARIYKNHFEQALMILIDNAVKYSRKEKKIAINLSVTGKQEAI
CNGIIICF_01748    VLREDFIFTWQSENPKTIARIYKNHFEQALMILIDNAVKYSRKEKKIAINLSVTGKQEAI
IKGFMBIA_01747    VLREDFIFTWQSENPKTIARIYKNHFEQALMILIDNAVKYSRKEKKIAINLSVTGKQEAI
DNELCKEF_00023    VLREDFIFTWQSENPKTIARIYKNHFEQALMILIDNAVKYSRKEKKIAINLSVTGKQEAI
LCNJLCPO_00752    VLREDFIFTWQSENPKTIARIYKNHFEQALMILIDNAVKYSRKEKKIAINLSVTGKQEAI
                  ************************************************************

SPy_0336          VRVQDKGEGISKEDIEHIFERFYRTDKSRNRTSTQAGLGIGLSILKQIVDGYHLQMKVES
SpyM3_0245        VRVQDKGEGISKEDIEHIFERFYRTDKSRNRTSTQAVLGIGLSILKQIVDGYHLQMKVES
CNGIIICF_01748    VRVQDKGEGISKEDIEHIFERFYRTDKSRNRTSTQAGLGIGLSILKQIVDGYHLQMKVES
IKGFMBIA_01747    VRVQDKGEGISKEDIEHIFERFYRTDKSRNRTSTQAGLGIGLSILKQIVDGYHLQMKVES
DNELCKEF_00023    VRVQDKGEGISKEDIEHIFERFYRTDKSRNRTSTQAGLGIGLSILKQIVDGYHLQMKVES
LCNJLCPO_00752    VRVQDKGEGISKEDIEHIFERFYRTDKSRNRTSTQAGLGIGLSILKQIVDGYHLQMKVES
                  ************************************ ***********************

SPy_0336             ELNEGSVFILHIPLAQSKES           M1GAS    (SPyxxxx)
SpyM3_0245           ELNEGSVFILHIPLAQSKES           MGAS315  (SPyM3_xxxx)
CNGIIICF_01748       ELNEGSVFILHIPLAQSKES           5448     (CNGIIICF_xxxxx)
IKGFMBIA_01747       ELNEGSVFILHIPLAQSKES           8157     (IKGFMBIA_xxxxx)
DNELCKEF_00023       ELNEGSVFILHIPLAQSKES           5626     (DNELCKEF_xxxxx)
LCNJLCPO_00752       ELNEGSVFILHIPLAQSKES           8003     (LCNJLCPO_xxxxx)
                     ********************
```

Fig. S7. Mutation within *covS* gene in 8003 strain results in T_{214}P substitution. Amino acid alignment of the indicated four GAS strains used in this study.

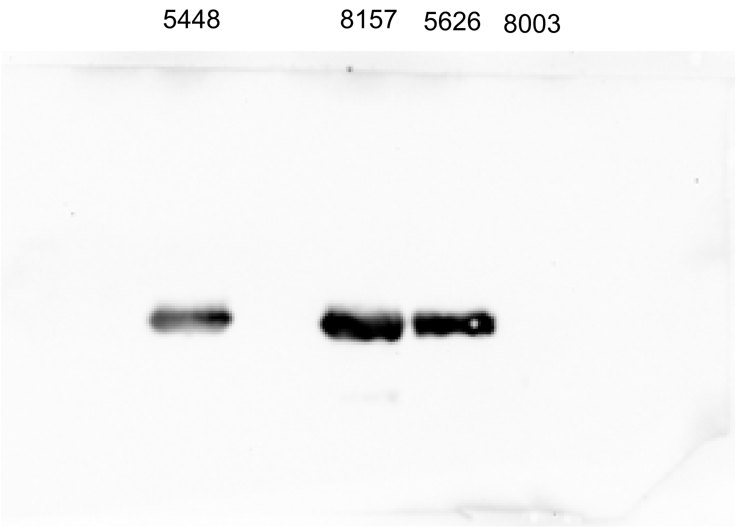

Fig. S8. Original SpeB blot as displayed in Fig. 2

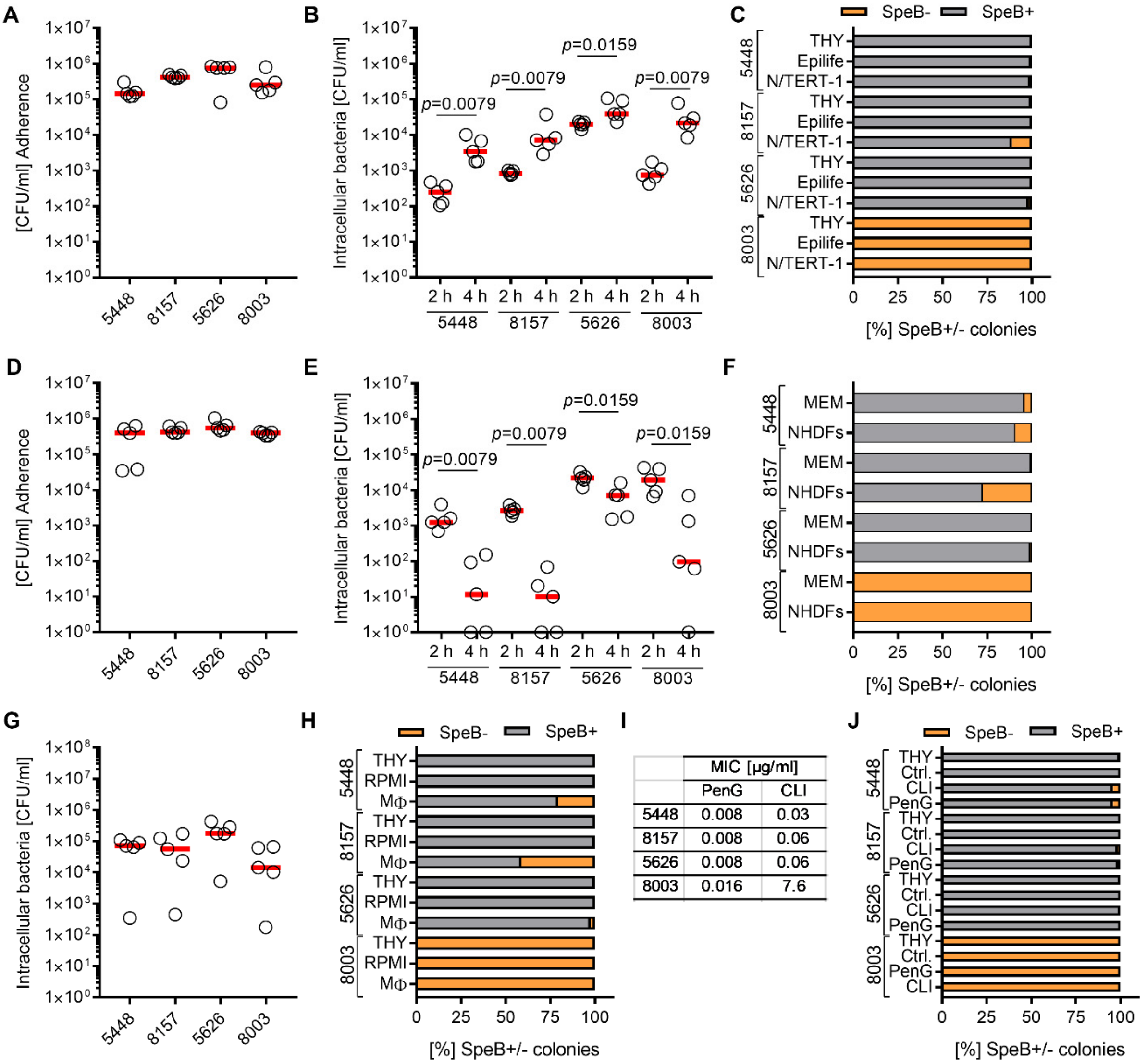

	MIC [µg/ml]	
	PenG	CLI
5448	0.008	0.03
8157	0.008	0.06
5626	0.008	0.06
8003	0.016	7.6

Fig. S9. SpeB phenotype in GAS co-culture with keratinocytes, fibroblasts, and macrophages. Extracellular (**A**) and intracellular (**B**) bacterial numbers recovered from keratinocytes after indicated time points of infection. Each dot represents one independent experiment. The horizontal lines denote median values (n=5). (**C**) Assessment of SpeB positivity/negativity of indicated strains via casein agar assay recovered after 4 h of intracellular passage in keratinocytes. Mean percentage from five independent experiments is shown (n=5). Extracellular (**D**) and intracellular (**E**) bacterial numbers recovered from human primary fibroblasts after indicated time points of infection. Each dot represents one independent experiment. The horizontal lines denote median values (n=5). (**F**) Assessment of SpeB positivity/negativity of indicated strains via casein agar assay recovered after 2 h of intracellular passage in fibroblasts. Mean percentage from five independent experiments is shown (n=5). (**G**) Intracellular bacterial counts after 2 h of macrophage infection. Each dot represents an experiment with macrophages from one donor. The horizontal lines denote median values (n=5). (**H**) Assessment of SpeB positivity/negativity of indicated strains recovered from primary human macrophages shown in (**G**). Mean percentage of SpeB+ and SpeB- clones from five independent experiments are shown (n=5). Bacteria incubated in EpiLife, RPMI, and THY media served as controls. (**I**) Penicillin G (PenG) and clindamycin (CLI) minimal inhibitory concentrations (MICs) of indicated

GAS strains (n=4). (**K**) Distribution of SpeB+ and SpeB- clones post antibiotic treatment. Displayed are mean [%] of four independent experiments (n=4). The level of significance was determined using Kruskal-Wallis test with Dunn´s multiple comparison post-test.

A

```
1      MNKKKLGVRLLSLLALGGFVLANPVFADQNFARNEKEAKDSAITFIQKSAAIKAGARSAE
61     DIKLDKVNLGGELSGSNMYVYNISTGGFVIVSGDKRSPEILGYSTSGSFDANGKENIASF
121    MESYVEQIKENKKLDTTYAGTAEIKQPVVKSLLDSKGIHYNQGNPYNLLTPVIEKVKPGE
181    QSFVGQHAATGCVATATAQIMKYHNYPNKGLKDYTYTLSSNNPYFNHPKNLFAAISTRQY
241    NWNNILPTYSGRESNVQKMAISELMADVGISVDMDYGPSSGSAGSSRVQRALKENFGYNQ
301    SVHQINRGDFSKQDWEAQIDKELSQNQPVYYQGVGKVGGHAFVIDGADGRNFYHVNWGWG
361    GVSDGFFRLDALNPSALGTGGGAGGFNGYQSAVVGIKP
```

B

SEQUENCE	START POSITION	END POSITION	LENGTH
DSAITFIQK	40	48	9
SAEDIKLDK	58	66	9
SPEILGYSTSGSFDANGK	97	114	18
ENIASFMESYVEQIK	115	129	15
ENIASFMESYVEQIKENK	115	132	18
KLDTTYAGTAEIK	133	145	13
LDTTYAGTAEIK	134	145	12
GIHYNQGNPYNLLTPVIEK	157	175	19
YHNYPNK	203	209	7
DYTYTLSSNNPYFNHPK	213	229	17
NLFAAISTR	230	238	9
QYNWNNILPTYSGR	239	252	14
ALKENFGYNQSVHQINR	291	307	17
ENFGYNQSVHQINR	294	307	14
QDWEAQIDK	313	321	9
QDWEAQIDKELSQNQPVYYQGVGK	313	336	24
ELSQNQPVYYQGVGK	322	336	15
VGGHAFVIDGADGR	337	350	14
LDALNPSALGTGGGAGGFNGYQSAVVGIKP	369	398	30

Fig. S10. SpeB single colony analysis. (**A**) Fasta sequence of SpeB-zymogene with SpeB tryptic peptides (green), detected by mass spectrometry in the single colony analysis. (**B**) SpeB tryptic peptides detected by mass spectrometry in the single colony analysis. The pro-domain is depicted in red whereas the sequence for mature SpeB is indicated in black.

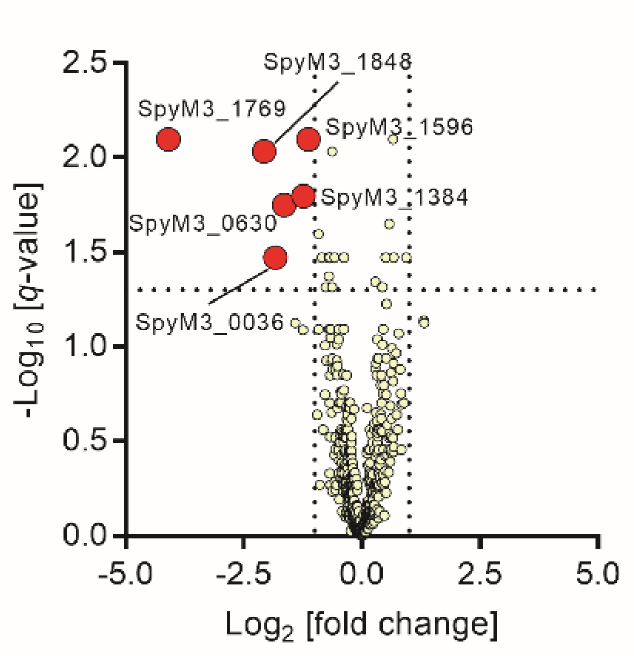

Fig. S11. Single colony proteome analysis of GAS 8003. Volcano plot displaying significant differences of single colony proteome post neutrophils infection compared to the THY control. Original data is displayed in Supplemental Table 3.

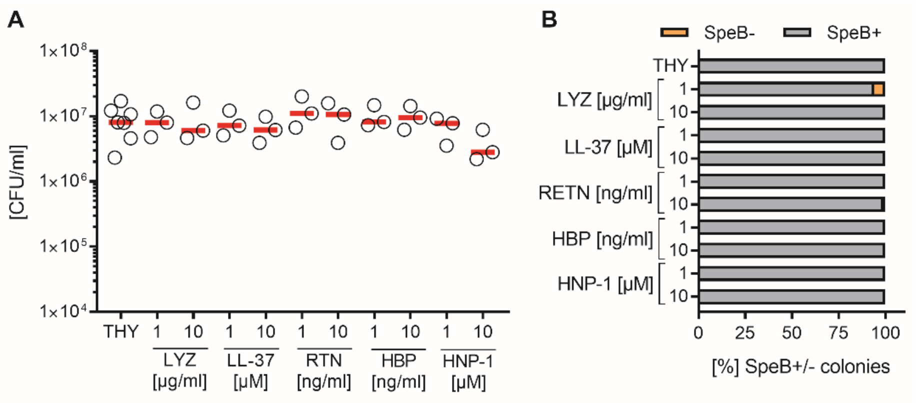

Fig. S12. The impact of neutrophil derived peptides/proteins on group A streptococcal SpeB secretion. 5448 GAS strain was exposed to indicated concentrations of proteins/peptides for 3 h and bacterial viability (**A**) and SpeB secretion (**B**) were assessed. (**A**) Each dot represents one independent experiment. The horizontal lines denote median values (n≥3). (**B**) Mean percentage from three independent experiments (shown in A) is displayed (LYZ, lysozyme; RTN, resistin; HBP, heparin binding protein; HNP-1, human neutrophil peptide 1).

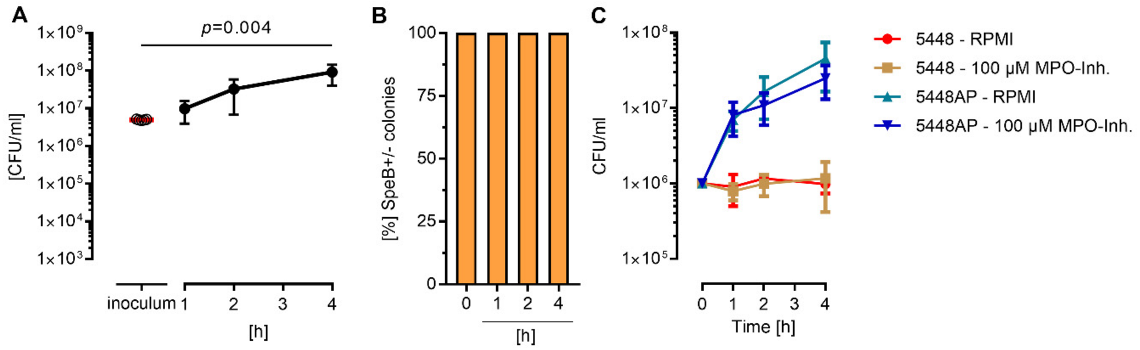

Fig. S13. 5448AP survives intracellular neutrophil passage. (***A***) Human primary neutrophils were infected GAS strain 5448AP and intracellular bacteria were determined by plating serial dilution of neutrophil lysates on casein agar plates post indicated time points. Dots represent the median value ±range of independent experiments with five donors (n=5). (***B***) Assessment of SpeB positivity/negativity of 5448AP strain recovered from primary human neutrophils shown in (***A***) (n=5). All 5448AP clones remained negative (orange). The level of significance between the groups of all experiments was determined using Kruskal Wallis test with Dunn´s posttest. (***C***) Control experiment confirming that 100 µM MPO-inhibitor had no effect on bacterial growth (n=3).

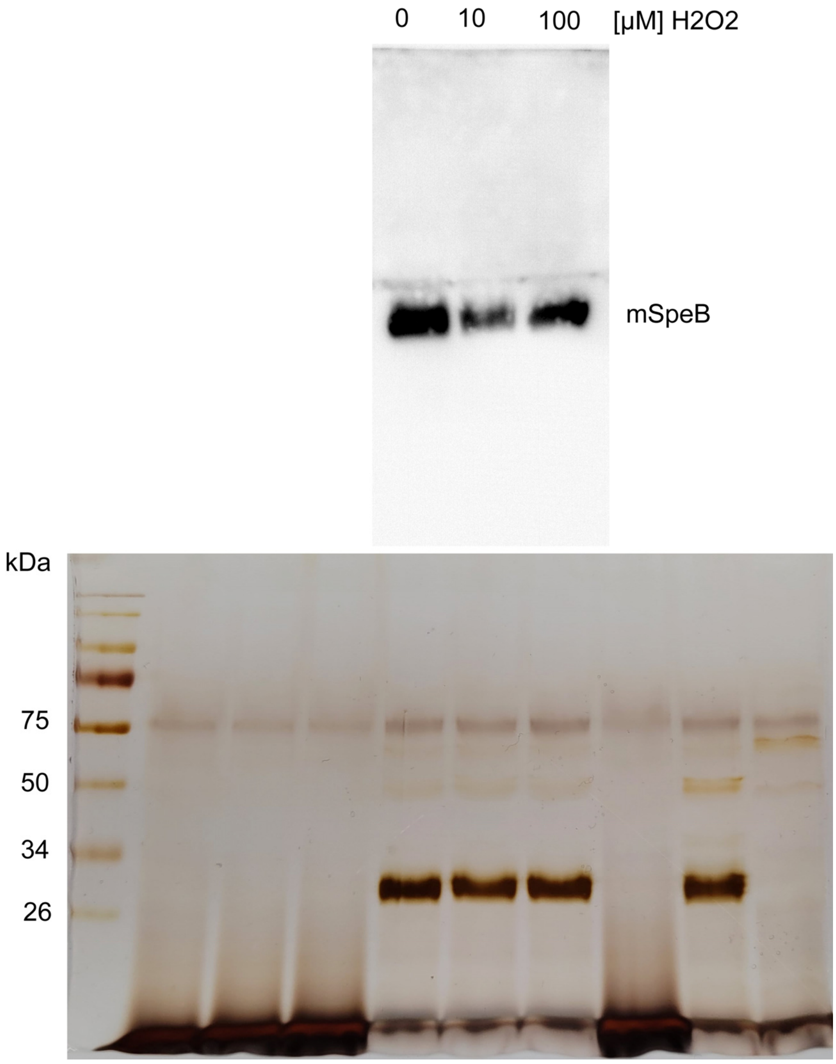

Fig. S14. Original Western blot (upper panel) and silver staining of the loading control (lower panel) of GAS 5448 supernatants post exposure to indicated concentrations of H_2O_2.

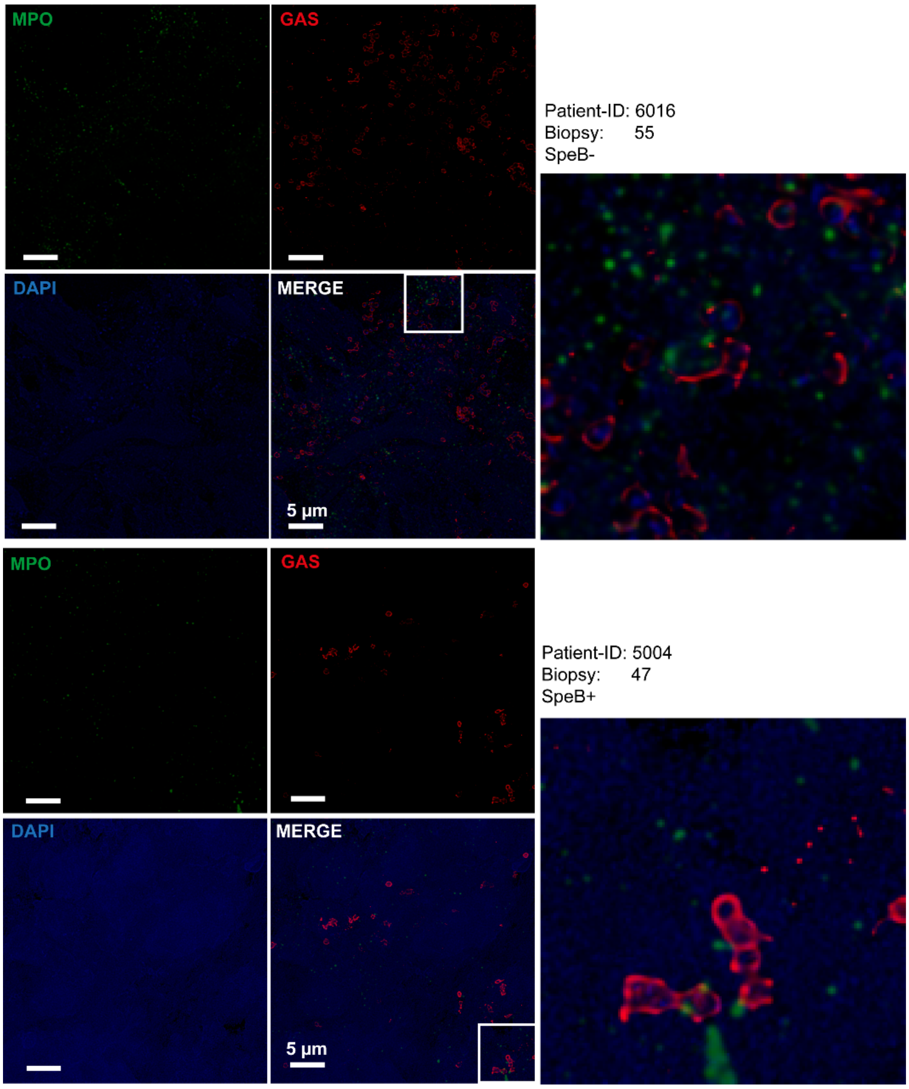

Fig. S15. Increased levels of MPO in patient tissue biopsies associated with SpeB^{-} GAS. Representative immunofluorescence micrographs of the distribution of MPO in patient biopsies.

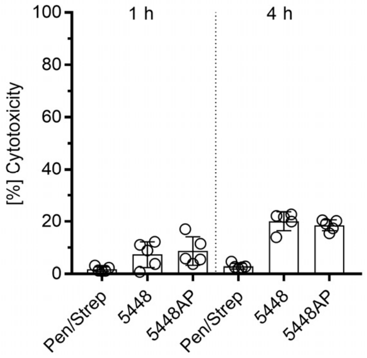

Fig. S16. Cytotoxicity induced by bacterial infections towards human primary neutrophils. Mean values ± s.d. from five donors (n=5) are shown. Each dot represents one donor.

Materials and Methods

Whole genome sequencing and data processing. DNA extraction from GAS strains 5448, 5626, 8003, and 8157, whole genome sequencing, and analyses were performed as previously described [14]. Briefly, Bacterial DNA was purified utilizing the DNeasy Blood & Tissue kit (Qiagen). Sequencing libraries were prepared using the NEBNext Ultra II DNA Library Prep Kit (NEB). Genomes were sequenced using Illumina MiSeq (2x300 bp). Sequencing data are deposited in the European Nucleotide Archive (ENA database) under the BioProject PRJNA524111 [3]. Raw reads were processed in paired-end mode by fastq-mcf version 1.05. Processed reads were assembled using SPAdes (version 3.11.1) de novo genome assembler software [2] in careful mode. Contigs with a length less than 200 bp or average coverage lower than 15 were discarded. Average contig coverage was determined by first mapping processed reads to the assembled contigs utilizing Bowtie2 (version 2.3.2) determining the per base coverage using the SAMtools (version 1.8) [11] and calculating the mean coverage for each contig. All assemblies were annotated utilizing Prokka genome annotation software (version 1.4) [13]. Gene comparisons are based on orthologous genes identified by the Proteinortho software (version 5.16b) [8]. Processed reads of 5448 and 8157 strains were mapped to the *S. pyogenes* M1GAS (*emm*1) reference genome and of 5626 and 8003 strains to MGAS315 (*emm*3) genome using Bowtie2 [6]. Variants were called using mpileup part of BCFtools (version 1.8) [10]. Variants were filtered out by a minimum SNP and INDEL distance of 10 bp, variant quality of 30, coverage of 50, mapping quality of 40 and a Z-score of 1.96 [5].

The genomes of GAS strains (Figure 1) were retrieved from the European Nucleotide Archive in FASTA format [9]. As reference strain, assembly GCF_000006785.2_ASM678v2 from the National Center for Biotechnology was used. The gene that encodes *covS* corresponds to the protein WP_002991036.1, *covR* corresponds to protein WP_002991052.1, and *ropB* corresponds to WP_002982409.1 in the reference strain. The reference protein sequences for CovS, CovR and RopB were searched in the genomes of strains using NCBI-BLAST version 2.9.0+ [1,4]. In case of CovR and RopB, the protein sequence in reference strains was extracted from the first BLAST hit, respectively. For CovS, the BLAST output showed that SPY2001 has a frame-shift causing deletion. Thus, cdbfasta tools version 0.99 (https://github.com/gpertea/cdbfasta, last accessed on February 18th 2021) were used to excise the corresponding coding sequence of BLAST hits from the strain genomes. For CovS, we performed a multiple

protein sequence alignment with CLUSTAL version 2.1 [15]. Subsequently, a translation of the aligned sequences was performed with AliView version 1.26 [7].

Protein extraction, LC-MS/MS analyses, and data processing of single GAS colonies and neutrophil secretome. Single SpeB$^+$ and/or SpeB$^-$ colonies, as assessed by casein agar assay, were transferred into tubes containing 200 µl 50 mM triethylammonium bicarbonate (TEAB) lysis buffer and 100 µl Lysing matrix B (MP Biomedicals). Bacteria were disrupted by bead beating (6 m/s, 5×20 s; 60 s ice incubation between the cycles) via FastPrep-24 5G (MP Biomedicals). Protein-containing supernatant was separated from bacterial debris via centrifugation (10 min, 10,000×g, 4°C). Protein concentrations were determined using BCA-assay (Thermo Fisher Scientific). 2.5 µg of total protein was reduced (5 mM Tris(2 carboxyethyl)phosphin [TCEP]; 45 min; 65°C), alkylated (10 mM iodoacetamide [IAA]; 15 min; room temperature; dark), and an in-solution digest with 250 ng of trypsin was performed (18 h; 37°C). Samples were concentrated and desalted via ZipTips (C18; Millipore), dried, and stored at -80°C until further use.

Samples were measured on a QExactive mass spectrometer coupled to an EASY nLC-1000 liquid chromatography system. Samples were loaded on an in-house packed column (ReproSil-Pur 120 C18-AQ, 3 µm) of 20 cm length and 100 µm inner diameter. Peptides were eluted by a non-linear 86 min gradient from 2% to 99% solvent B (acetonitrile with 0.1% acetic acid). The overview scans (MS1) covered a mass range of 300–1650 m/z at a resolution of 70,000 (at 200 m/z). The 12 most abundant precursors were selected for HCD fragmentation at NCE 27.5 with an AGC target of 1e5 and an under-fill ratio of 5%. Dynamic exclusion was set to 30 s, lock mass correction was enabled, and ions with unknown charge of one or higher than six were excluded from fragmentation.

MaxQuant version 1.6.10.43 and ncbi genome assembly data base id=233599 (M1GAS) and id=299846 (MGAS315) were used for protein identification. The minimal number of unique peptides per protein group was set to 2 to be considered as identified. Oxidation (M) was considered as a variable and carbamidomethyl (C) as a fixed modification. Data were analyzed using Perseus version 1.6.10.43. Quantitative values were $\log_2$ transformed and the resulting values were filtered based on the following criteria: only identified by site, reverse, potential contamination, quantified in at least 75% of biological

replicates per group. Differential expression of bacterial proteins was determined via Student´s *t* test with Benjamini-Hochberg FDR correction. Proteins were considered to be significantly differentially expressed if *q*-value was <0.01 and log_2 fold change was ≥1. The principle component analysis (PCA) of the scaled data was carried out using the ClustVis web tool [12].

Neutrophil secretome profiling: 500 µl neutrophil supernatant was reduced with TCEP (5 mM, 45 min, 65°C) and alkylated with IAA (10 mM, 20 min, room temperature, dark). Five microliters of SP3 beads (hydrophobic: Sera-Mag Speedbeads carboxylate-modified particles [GE Healthcare]; hydrophilic: Speedbead magnetic carboxylate-modified particles [GE Healthcare]) were added to the sample and acetonitrile added to a final concentration of 70% (v/v) and incubated in a thermomixer (5 min, 24 °C, 900 rpm). Tubes were placed in a magnetic rack to collect the beads and the supernatant was removed. Beads were washed with 80% (v/v) ethanol twice and air dried. Proteins were digested by adding 25 µl digestion buffer (50 mM TEAB) containing 100 ng of trypsin followed by 30 s sonication in a water bath to disaggregate the beads and an incubation at 37°C for 18 h. Beads were removed by centrifugation (20,000 g, 1 min) and by placing the tubes in a magnetic rack to transfer the supernatant to a glass vial. The supernatants were dried by vacuum centrifugation and peptides were reconstituted in 12 µl 0.1% acetic acid in water containing iRT peptides.

Generated tryptic peptides were analyzed by LC-MS/MS. Therefore, an EASY nLC 1000 (Thermo Fischer) was coupled to an QExactive mass spectrometer (Thermo Fisher). Peptides were loaded onto in house packed fused silica columns of 20 cm length and an inner diameter of 75 µm, filled with Dr. Maisch ReproSil Pur 120 C18-AQ 1.9 µm (Dr. Maisch). Peptides were eluted using a non-linear binary gradient of 86 min from 2% to 99% solvent B (0.1% acetic acid in acetonitrile) in solvent A (0.1% acetic acid). The overview scans (MS1) covered a mass range of 300–1650 m/z at a resolution of 70,000 (at 200 m/z). The 10 most abundant precursors were selected for HCD fragmentation at NCE 27.5 with an AGC target of 1e5 and an under-fill ratio of 10%. Dynamic exclusion was set to 30 s, lock mass correction was enabled, and ions with unknown charge, charge of one or higher than six were excluded from fragmentation.

MaxQuant version 1.6.17.0 and ncbi genome assembly data base id=233599 (M1GAS) and UniProt human reference proteome (downloaded 20190715) were used for protein identification. The minimal number of

unique peptides per protein group was set to 2 to be considered as identified. Oxidation (M) was considered as a variable and carbamidomethyl (C) as a fixed modification. Data were analyzed using Perseus version 1.6.14.0. Quantitative values were $\log_2$ transformed and the resulting values were filtered based on the following criteria: only identified by site, reverse, potential contamination, quantified in at least 4 of 5 biological replicates per group. LFQ intensities were normalized to the intensity of the spiked iRT. Differential expression of bacterial proteins was determined via Student´s *t* test with Benjamini-Hochberg FDR correction. Proteins were considered to be significantly differentially expressed if p-value was <0.05 and $\log_2$ fold change was ≥ 1.

Cytotoxicity LDH release assay. Cytotoxicity was determined by measurement of the LDH activity via CytoTox 96 Non-Radio Kit (Promega) according to manufacturer's guidelines.

References

1. Altschul S.F., Gish W., Miller W., Myers E.W. and Lipman D.J. Basic local alignment search tool. J Mol Biol 215(3):403-410, 1990.
2. Bankevich A., Nurk S., Antipov D., Gurevich A.A., Dvorkin M., Kulikov A.S., Lesin V.M., Nikolenko S.I., Pham S., Prjibelski A.D., Pyshkin A.V., Sirotkin A.V., Vyahhi N., Tesler G., Alekseyev M.A. and Pevzner P.A. SPAdes: a new genome assembly algorithm and its applications to single-cell sequencing. J Comput Biol 19(5):455-477, 2012.
3. Bruun T., Rath E., Bruun Madsen M., Oppegaard O., Nekludov M., Arnell P., Karlsson Y., Babbar A., Bergey F., Itzek A., Hyldegaard O., Norrby-Teglund A., Skrede S. and Group I.S. Risk factors and Predictors of Mortality in Streptococcal Necrotizing Soft-Tissue Infections: A Multicenter Prospective Study. Clin Infect Dis, 2020.
4. Camacho C., Coulouris G., Avagyan V., Ma N., Papadopoulos J., Bealer K. and Madden T.L. BLAST+: architecture and applications. BMC Bioinformatics 10:421, 2009.
5. Kaas R.S., Leekitcharoenphon P., Aarestrup F.M. and Lund O. Solving the problem of comparing whole bacterial genomes across different sequencing platforms. PLoS One 9(8):e104984, 2014.
6. Langmead B. and Salzberg S.L. Fast gapped-read alignment with Bowtie 2. Nat Methods 9(4):357-359, 2012.
7. Larsson A. AliView: a fast and lightweight alignment viewer and editor for large datasets. Bioinformatics 30(22):3276-3278, 2014.
8. Lechner M., Findeiss S., Steiner L., Marz M., Stadler P.F. and Prohaska S.J. Proteinortho: detection of (co-)orthologs in large-scale analysis. BMC Bioinformatics 12:124, 2011.
9. Leinonen R., Akhtar R., Birney E., Bower L., Cerdeno-Tarraga A., Cheng Y., Cleland I., Faruque N., Goodgame N., Gibson R., Hoad G., Jang M., Pakseresht N., Plaister S., Radhakrishnan R., Reddy K., Sobhany S., Ten Hoopen P., Vaughan R., Zalunin V. and Cochrane G. The European Nucleotide Archive. Nucleic Acids Res 39(Database issue):D28-31, 2011.
10. Li H. A statistical framework for SNP calling, mutation discovery, association mapping and population genetical parameter estimation from sequencing data. Bioinformatics 27(21):2987-2993, 2011.
11. Li H., Handsaker B., Wysoker A., Fennell T., Ruan J., Homer N., Marth G., Abecasis G., Durbin R. and Genome Project Data Processing S. The Sequence Alignment/Map format and SAMtools. Bioinformatics 25(16):2078-2079, 2009.
12. Metsalu T. and Vilo J. ClustVis: a web tool for visualizing clustering of multivariate data using Principal Component Analysis and heatmap. Nucleic Acids Res 43(W1):W566-570, 2015.
13. Seemann T. Prokka: rapid prokaryotic genome annotation. Bioinformatics 30(14):2068-2069, 2014.
14. Siemens N., Oehmcke-Hecht S., Hossmann J., Skorka S.B., Nijhuis R.H.T., Ruppen C., Skrede S., Rohde M., Schultz D., Lalk M., Itzek A., Pieper D.H., van den Bout C.J., Claas E.C.J., Kuijper E.J., Mauritz R., Sendi P., Wunderink H.F. and Norrby-Teglund A. Prothrombotic and Proinflammatory Activities of the beta-Hemolytic Group B Streptococcal Pigment. J Innate Immun:1-13, 2019.
15. Thompson J.D., Higgins D.G. and Gibson T.J. CLUSTAL W: improving the sensitivity of progressive multiple sequence alignment through sequence weighting, position-specific gap penalties and weight matrix choice. Nucleic Acids Res 22(22):4673-4680, 1994.
